# The Future of Neurotoxicology: A Neuroelectrophysiological Viewpoint

**DOI:** 10.3389/ftox.2021.729788

**Published:** 2021-12-14

**Authors:** David W. Herr

**Affiliations:** Neurological and Endocrine Toxicology Branch, Public Health and Integrated Toxicology Division, CPHEA/ORD, U.S. Environmental Protection Agency, Washington, NC, United States

**Keywords:** neurotoxicololgy, neurophysiology, adverse outcome pathway, neural networks, mechanistic

## Abstract

Neuroelectrophysiology is an old science, dating to the 18th century when electrical activity in nerves was discovered. Such discoveries have led to a variety of neurophysiological techniques, ranging from basic neuroscience to clinical applications. These clinical applications allow assessment of complex neurological functions such as (but not limited to) sensory perception (vision, hearing, somatosensory function), and muscle function. The ability to use similar techniques in both humans and animal models increases the ability to perform mechanistic research to investigate neurological problems. Good animal to human homology of many neurophysiological systems facilitates interpretation of data to provide cause-effect linkages to epidemiological findings. Mechanistic cellular research to screen for toxicity often includes gaps between cellular and whole animal/person neurophysiological changes, preventing understanding of the complete function of the nervous system. Building Adverse Outcome Pathways (AOPs) will allow us to begin to identify brain regions, timelines, neurotransmitters, etc. that may be Key Events (KE) in the Adverse Outcomes (AO). This requires an integrated strategy, from *in vitro* to *in vivo* (and hypothesis generation, testing, revision). Scientists need to determine intermediate levels of nervous system organization that are related to an AO and work both upstream and downstream using mechanistic approaches. Possibly more than any other organ, the brain will require networks of pathways/AOPs to allow sufficient predictive accuracy. Advancements in neurobiological techniques should be incorporated into these AOP-base neurotoxicological assessments, including interactions between many regions of the brain simultaneously. Coupled with advancements in optogenetic manipulation, complex functions of the nervous system (such as acquisition, attention, sensory perception, etc.) can be examined in real time. The integration of neurophysiological changes with changes in gene/protein expression can begin to provide the mechanistic underpinnings for biological changes. Establishment of linkages between changes in cellular physiology and those at the level of the AO will allow construction of biological pathways (AOPs) and allow development of higher throughput assays to test for changes to critical physiological circuits. To allow mechanistic/predictive toxicology of the nervous system to be protective of human populations, neuroelectrophysiology has a critical role in our future.

## INTRODUCTION

A proposal of future directions for application of neuroelectrophysiological techniques in toxicology must define some of the current and future problems facing the science of neurotoxicology. Currently (and in the foreseeable future), two major emphases in the field of neurotoxicology are: 1) mechanistic information and 2) human cognitive alterations (including diseases such as attention deficit hyperactivity disorder (ADHD), autism, learning disabilities, motor and sensory deficits, etc.). Both areas can be advanced through the systematic collection of targeted functional mechanistic data based on known or presumed biological pathways (Adverse Outcome Pathways (AOPs) in the field of neurotoxicology). Neurophysiology has the ability to bridge mechanistic data and behavioral changes—a critical linkage. As indicated below, the collection of mechanistic data to understand the function of the nervous system has been occurring for a long time.

## PAST

When considering future directions for neuroelectrophysiology as applied to neurotoxicology, it is beneficial to consider the historical progression of advancements and how the area of neurophysiology has contributed to the fields of neuroscience. Electrophysiology has provided a valuable basic understanding of nervous system function in neurobiology and neurotoxicology for centuries. The history of neurophysiology has been detailed in more exhaustive reviews and is only summarized here (Collura, 1993; Muley et al., 2009; Piccolino, 1998; Verkhratsky and Parpura, 2014). In the 1660’s, Dr. Jan Swammerdam dissected a frog leg and discovered that muscle fiber contraction could be induced by stimulation of nerve fibers (Muley et al., 2009; Verkhratsky et al., 2006). In 1791, Luigi Galvani published seminal work regarding nerve-muscle preparations in a frog, leading to the understanding of stimulus-response and muscle contractions (Galvani, 1791; Muley et al., 2009; Piccolino, 1998; Verkhratsky et al., 2006), and proposed that accumulation of positive and negative charges along the surface of muscles and nerve fibers resulted in “animal electricity”. Also using a nerve-muscle preparation, Leopoldo Nobili recorded the first evidence of the involvement of electrical activity (Nobili, 1828), although he apparently failed to appreciate the intrinsic biological origin. In about 1848, a crude recording of an action potential was made by Emile du Bois-Reymond (du Bois-Reymond, 1848). A few years later, a measure of nerve conduction involved in producing muscular contraction was made by Hermann Ludwig Ferdinand von Helmholtz in 1850 (Helmholtz, 1850a; Helmholtz, 1850b), and published with graphics in 1852 (Helmholtz, 1852). The first “true” measure of nerve conduction velocity (NCV) was published in 1868 by Julius Bernstein (Bernstein, 1868; Bernstein, 1871), who also verified that an action potential involved a charge movement which exceeded the resting membrane potential. Continuing the evolution of knowledge relating to nerve electrical activity, the involvement of ions (potassium) in nerve currents was proposed in 1912 (Bernstein, 1912). The theory of local circuits was proposed by Ludimar Hermann (Hermann, 1872; Hermann, 1873) postulating that a nerve contained a conductive “core”, an insulating sheath, and an external fluid medium, and that an electrical disturbance would result in nearby portions of the nerve completing current loops. However, it was Charles Overton who demonstrated that sodium ions were involved in the action potential overshoot (Overton, 1902). The electrical activity in single sensory fibers, and the encoding of stimulation intensity in muscles as the firing rate of the sensory fibers was reported in 1926 (Adrian, 1926; Adrian and Zotterman, 1926). This finding formed the basis for explaining how intensity could be encoded in the “all-or-none” triggering of what are today known as action potentials. The development of the voltage clamp technique allowed researchers to accurately monitor current flow across neuronal membranes (Cole, 1949; Marmont, 1949). The involvement of ionic sodium in action potentials was finally described by Hodgkin and Katz in 1949 using giant squid axons (Hodgkin and Katz, 1949), leading to the development of the Hodgkin-Huxley model for the ionic generation of action potentials (Hodgkin et al., 1952; Hodgkin and Huxley, 1952). The Hodgkin-Huxley model, coupled with the invention of the patch clamp technique in 1976 (Neher and Sakmann, 1976), allowed ionic flux thorough single channels to be studied. After approximately 185 years, the biophysical basis for the “animal electricity” proposed by Galvani was defined in detail.

While the above discoveries were obtained in peripheral nerve/muscle preparations, electrical activity in the brain was also being investigated. Early investigations involved brain stimulation and observing motor responses, which initiated the science of mapping the regions of differing brain function (Hitzig and Fritsch, 1870). In 1875 and 1877, Richard Canton recorded electrical activity from the brains of animals (Caton, 1875, Caton, 1877). These recordings involved Electroencephalogram (EEG) changes in sleep and awake states, as well as responses to auditory and somatosensory stimulation. These latter experiments may represent the first recordings of sensory evoked responses. The impact of peripheral stimulation on cortical desynchronization and recording responses localized on the cortex surface of animals was reported by Adolf Beck in 1890 (Beck, 1890; Coenen and Zayachkivska, 2013). Spontaneous human EEG recordings were first published by Hans Berger in 1929, documenting the importance of electrical activity in the human brain (Berger, 1929). In 1932, Dietsch introduced Fourier analysis of the EEG signal, a process that is still in use today to describe different EEG waveform bandwidths (Dietsch, 1932). The use of multichannel EEG recordings allowed description of spatial and temporal variations (Adrian and Matthews, 1934). As the techniques evolved, applications of EEG monitoring to localization of various brain waves was developed. An early medical application of human EEG involved localization of epileptic seizures, for subsequent surgical treatment (Jasper et al., 1940). These examples briefly show the valuable historical contribution of *in vivo* neurophysiology to the world of biology and medicine.

## PRESENT

### Mechanistic Neuroelectrophysiology

Simultaneously with the advancement of knowledge of basic biological physiology, integration with pharmacology and toxicology was occurring. This, in turn, lead to investigations into the mechanism of action of various chemicals using neurophysiological techniques. In general, recordings can be made from single cells (allowing examination of cell action potentials, ion flux through channels) or field potentials from the extracellular space (that may represent activity from multiple neurons). Some basic descriptions of methods mentioned below and considerations in their application are summarized in Table 1.

Again, while the scope of this manuscript prevents exhaustive methodological and toxicological details, it is hoped the reader can use the examples provided as a basis for additional research. One of the first toxicological mechanistic studies was in 1857, when Claude Bernard used a neuromuscular preparation to show that curare interfered with neurotransmission at the level of the neuromuscular junction (Bernard, 1857; Gray, 1947). Mechanistic investigations can also be exemplified by proof of dichlorodiphenyltrichloroethane’s (DDT) effects on action potentials, which involved alterations at the level of sodium and potassium channels (Shanes, 1949; Narahashi and Yamasaki, 1960; Narahashi and Haas, 1967; Narahashi and Haas, 1968). Other investigators used *in vitro* preparations to demonstrate that mercury or lead altered presynaptic neurotransmitter release, presumably by interfering with calcium function (Manalis and Cooper, 1973; Manalis and Cooper, 1975; Kober and Cooper, 1976). *In vivo* neurophysiological measures of excitability and plasticity (indicated by population spikes, short- and long-term potentiation, kindling, etc …) (Goddard et al., 1969; Lømo, 1971; Racine et al., 1972; Bliss and Lømo, 1973; Douglas and Goddard, 1975; Racine et al., 1983) opened the door for investigations related to network function and plasticity. Studies using these techniques illustrated the effect of lindane on GABA-mediated inhibition in the hippocampus (Joy and Albertson, 1985; Joy and Albertson, 1987; Joy et al., 1995). The effects of lindane, dieldrin, and endosulfan on increasing neuronal network excitability were also investigated using seizure models such as kindling (Joy et al., 1980; Joy et al., 1982; Gilbert, 1992, Gilbert, 1995; Gilbert and Mack, 1995). An increased decay of long-term potentiation was shown following long-term exposure to lead in drinking water (Gilbert and Mack, 1998). A paradigm known as paired-pulse inhibition was used to following treatment with cismethrin (Type I) or fenvalerate or deltamethrin (Type II) pyrethroids, and indicated changes in sodium channel kinetics supported the data better than changes in GABAergic function—providing a mechanistic basis for the altered network physiology (Gilbert et al., 1989). Although some authors have expressed limitations with respect to extrapolations to behavioral changes (Hölscher, 1997), neurophysiological phenomena such as long-term potentiation/depression have been used as models to study the neural plasticity associated with biological constructs such as learning and memory (Lynch, 2004; Nicoll, 2017; Abraham et al., 2019). In conjunction with methods discussed later in this paper, these types of studies addressed issues such as *in vitro* to *in vivo* extrapolation and the need to examine higher cognitive function, which are still pressing issues of concern in toxicology.

### Evoked Potentials With Clinical Applications

While less mechanistic in nature than the above examples, other neurophysiological procedures, such as evoked potentials (EPs) have contributed to both the fields of neurobiology and toxicology. To record this type of neurophysiological response, a stimulus is presented to the test subject, and the time-locked signal of the nervous system is recorded (often involving signal averaging). As mentioned previously, this type of procedure may have first been used by Canton (Brazier, 1984; Caton, 1875; Caton, 1877). Evoked potentials have served as a neurological technique to characterize and help localize the neuroanatomical basis of neurotoxicity.

Assessment of changes in the peripheral nervous and muscular system has served to characterize the neurotoxicity of numerous chemicals. Examination of the nerve-muscle physiology can involve electromyographic (EMG) examination. These tests have been shown to be sensitive to several types of environmental toxicants or experimental manipulations, and many of these procedures have been described by others (Arezzo et al., 2011; Howard, 2013; McNeil et al., 2013) and will only be briefly described here. Perhaps the simplest test is of spontaneous EMG activity, which often increases in the presence of neurotoxicity or disease (Hnik et al., 1982; Ross and Lawhorn, 1990; Daube and Rubin, 2009). Stimulation of motor nerves allows recording of muscle action potentials (M-wave), which can be altered in the presence of neurotoxicity (Ross and Lawhorn, 1990). The elapsed time from the stimulus to the recorded neurophysiological response is known as the distal latency, and reflects the conduction velocity of the motor fibers in the stimulated nerve when the M-wave is recorded (Mallik and Weir, 2005). This measure has been shown to be a sensitive measure for intoxication with 2,5-hexanedione (Nachtman and Couri, 1984). A method known as single fiber jitter testing (voluntary or stimulation methods), measures the variability in neuromuscular transmission time between successive muscle action potentials (Stålberg and Sanders, 1981; Stålberg and Sonoo, 1994). Changes in neuromuscular jitter have been reported in mice after treatment with the organophosphates (OPs) mipafox or ecothiopate (Kelly et al., 1994). Decrements in muscle action potential amplitude after repetitive nerve stimulation (RNS) have been shown after treatment with the OPs dimethoate (Dongren et al., 1999), fenvalerate, or phoxim (Yang et al., 2001) or the depolarizing neuromuscular drug decamethonium (Finley et al., 2009). As reviewed by Le Quesne, changes in EMG responses have been shown following exposure to acrylamide, lead, organophosphates, or hexacarbons (Le Quesne, 1978; Hnik et al., 1982). Additional measures such as the F-wave (antidromic propagation along motor axons to the motor neuron cell, with subsequent firing and production of a small M-wave) (Panayiotopoulos and Chroni, 1996) and the H-reflex (stimulation of sensory fibers which then activate motor neurons at level of spinal cord, with subsequent firing of motor neurons and eliciting a M-Wave) (Cliffer et al., 1998b; Tucker et al., 2005) are also possible, although they may be technically challenging (Mattsson et al., 1984). The H-reflex may help disassociate sensory vs motor changes, due to the involvement of the sensory component, which should be absent/reduced in the M- or F-responses (Hamers et al., 1991; Cliffer et al., 1998a).

Often closely associated with electromyography is the assessment of peripheral nerve function (Arezzo et al., 2011). Nerve conduction velocity is the measure of the speed of action potential propagation along a nerve (stimulation and recording are along the nerve itself) and generally reflects the conduction speed of the largest diameter axons (Stålberg and Erdem, 2000). Changes in myelin will alter the normal saltatory conduction and change the NCV, whereas damage/death of the nerve will tend to alter the amplitude of the nerve action potential (Kimura, 1984; Hamers et al., 1991; Mattsson et al., 1992). Because the size and shape of a nerve action potential reflects the different constituent nerve fibers of the stimulated nerve (Gasser and Erlanger, 1927), changes in the distribution of conduction velocities may provide insight whether different types of nerve fibers are preferentially affected by a toxicant (Dorfman, 1984; Caccia et al., 1993; Ruijten et al., 1993). Examples of compounds that have been shown to alter nerve conduction and/or nerve action potentials include hexane (Howd et al., 1983; Rebert and Sorenson, 1983; Nylén et al., 1994), taxol (Cavaletti et al., 1995; Leandri et al., 2012), carbon disulfide (Herr et al., 1998), nitrile chemicals (Gagnaire and Marignac, 1999), hexachlorophene (DeJesus et al., 1978), and cisplatin (Rebert et al., 1984; Thompson et al., 1984; De Koning et al., 1987).

Stimulation of different sensory systems can be used to assess changes in somatosensory, auditory, and visual function (Dyer, 1985). Because the parts of the nervous system that generate these sensory EPs are generally known (Rebert, 1983; Mattsson and Albee, 1988; Herr and Boyes, 1995), the changes in EPs can help with neuroanatomical localization of altered physiological response, and can be integrated into a toxicological profile that includes targeted histopathological investigations (Mattsson et al., 1989a; Ross, 1989; Mattsson et al., 1990; Mattsson et al., 1992; Morgan et al., 2004; Arezzo et al., 2011). An example for the visual system is the electroretinogram (ERG). This response includes components reflecting activity at the levels of the photoreceptors, the bipolar-Müeller cells, and for pattern stimuli, may include ganglion cell components (Baker Jr et al., 1988; see; Herr and Boyes, 1995 for review; Heynen and Van Norren, 1985; Maffei and Fiorentini, 1986; Miura et al., 2009). As a second example, auditory stimulation allows recording of the brainstem auditory evoked response. The EP consists of a series of peaks that include physiological responses from the cochlear hair cells, the auditory nerve, cochlear nucleus, superior olivary complex lateral lemniscus, inferior colliculus, medial geniculate nucleus, and can include the auditory cortex (see Herr and Boyes, 1995; Mattsson et al., 1992 for reviews). The use of analogous neurophysiological techniques between laboratory animals and humans can assist in extrapolating effects across species (Hudnell et al., 1990; Benignus et al., 1991; Boyes, 1994). Additionally, test guidelines have been developed for using these techniques in a neurotoxicological setting (United States Environmental Protection Agency, 1998a; United States Environmental Protection Agency, 1998b). These guidelines cover the evaluation of peripheral nerve function, NCV, and sensory evoked potentials in toxicological studies for submission to the U.S. E.P.A., and can be adapted for acute, chronic, or developmental studies. These types of EP tests have been used to assess somatosensory alterations produced by dichloroacetylene (Albee et al., 1997), carbonyl sulfide (Herr et al., 2007), toluene or *o*-cresyl (Mattsson et al., 1989b), or hexane (Rebert and Sorenson, 1983). Changes in the auditory system have been shown following treatment with jet fuel coupled with noise exposure (Fechter et al., 2007), carbonyl sulfide (Morgan et al., 2004; Sills et al., 2004; Herr et al., 2007), chlordimeform or developmental glutamate (Janssen et al., 1983; Janssen et al., 1991), or polychlorinated biphenyls (Lilienthal et al., 2011; Poon et al., 2011). Changes in the ERG have been reported after exposure to methanol (Eells et al., 1996; Eells et al., 2000), cholinesterase inhibition (Jones et al., 1995), or lead (Fox and Rubinstein, 1989). Additionally, changes in the central nervous system function of the visual system (Boyes, 1992) have been shown following treatment with trichloroethylene (Boyes et al., 2005), carbon disulfide (Herr et al., 1992), 3,3′-iminodipropionitrile (Herr et al., 1995), and carbaryl or propoxur (Mwanza et al., 2008). The utility of these EP methods to detect, help localize the site of neurological dysfunction, and applicability to human neurology assures that such methods will continue to be applied in the future.

### Neuroelectrophysiology and Cognitive Measures

Although largely in neuroscience and clinical settings, neurophysiological methods have also been used to study neural generators involved in cognitive processing of external stimuli using both methods based on recording EEG and/or evoked responses. The amount of literature regarding source localization (Grech et al., 2008; Asadzadeh et al., 2020) involving methods such as dipole source modeling (Wood, 1982; Koles, 1998), coherence measures (Nunez, 1995; Hoechstetter et al., 2004), neural networks (Abeyratne et al., 1991; Cui et al., 2019) and many other methods, is beyond the scope of this paper. However, such techniques (along with signal averaging) have been used to study associations between cognitive processing and neurophysiological responses. A negative peak recorded over the fronto-central regions of the brain at about 100 ms after stimulus delivery (N100) is proposed to be related to attentional processing, with the amplitude related to the salience of the stimuli (Haider et al., 1964; Hansen and Hillyard, 1980; Vogel and Luck, 2000). A negative potential at around 150 ms can be recorded after an incorrect response during tasks where the subject is required to identify a correct stimulus. It is recorded over the cingulate cortex and is thought to be a subconscious reflection of error monitoring (Falkenstein et al., 1991; Gehring et al., 1993; Carter et al., 1998). A negative potential recorded over the frontal cortex between 200–350 ms (N200) has been related to response inhibition, attention orientation, and error detection (Wijers et al., 1989; Jodo and Kayama, 1992). A well-studied positive potential occurring about 300 ms (P300) after a stimulus has been related to the “significance” of the stimulus, and has been related to constructs such as attention and working memory (Sutton et al., 1965; Verleger, 1988; Polich, 2007). These types of studies move the application of neurophysiology beyond neurotransmission and sensory perception, and into the realm of higher cognitive processing.

## FUTURE

**FIGURE 1 |:**
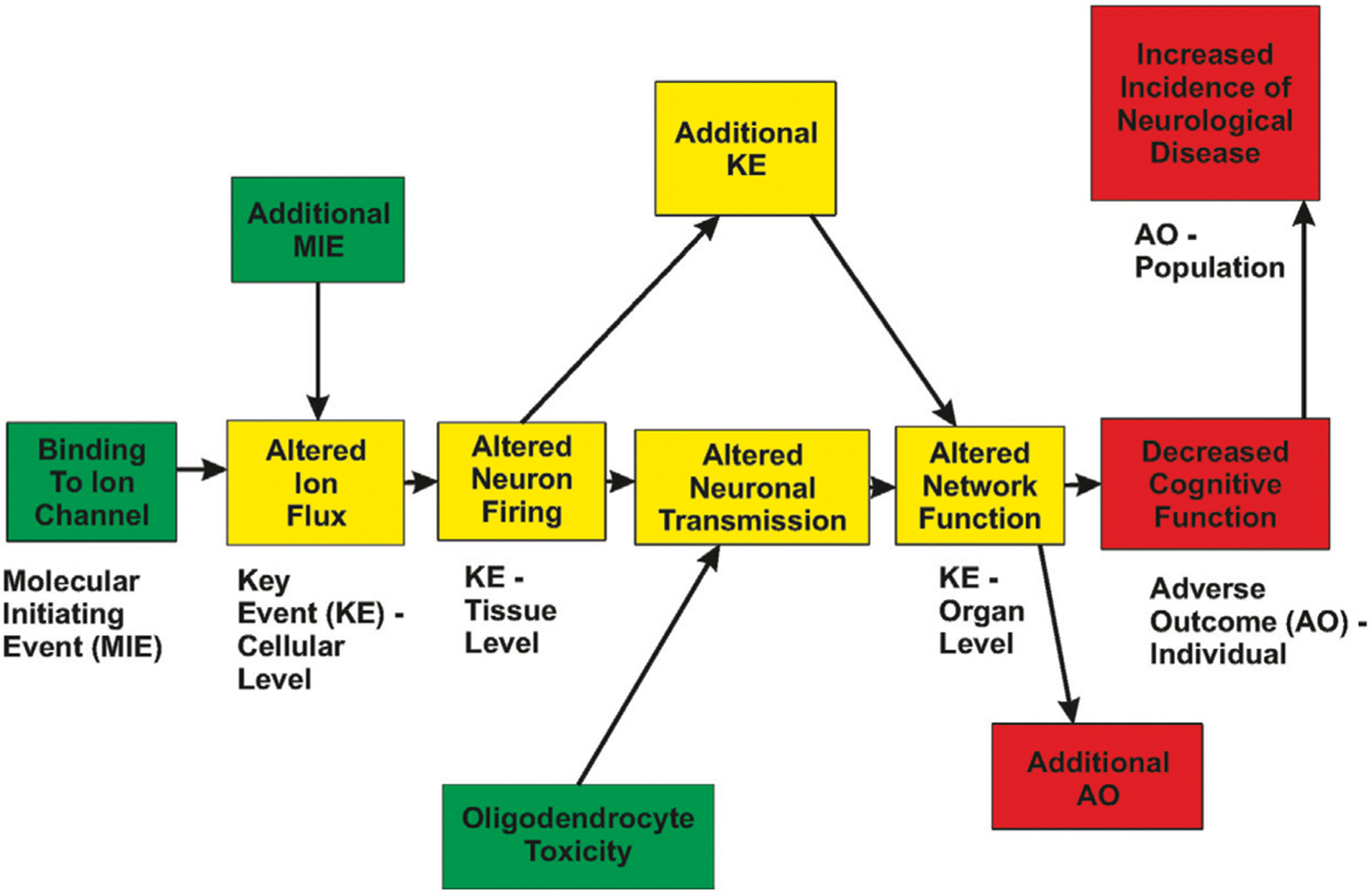
Hypothetical Adverse Outcome Pathway relationship. A xenobiotic interacts with biological tissue and results in a Molecular Initiating Event (MIE). This change in biology produces an alteration in a measurable Key Event (KE), which in turn, results in the subsequent change in additional Key Events. This progression leads to measurable changes at the cellular, tissue, and organ levels of biology. Note that multiple MIEs can impinge on a single KE, and KEs can interact in a network manner. Eventually, the biology is altered sufficiently to result in an Adverse Outcome (AO) that is of concern to society. Altered ion flux could be measured using patch clamp techniques, altered neuronal firing could be measured using multi-electrode arrays, altered network function could be measured using evoked potentials or EEG measures.

Future directions for neurophysiology will incorporate the generation of data for functional changes as integrated into presumed, or known, biological pathways. Advances in *in vitro* high-throughput screening coupled with the recommendation of the National Research Council (NRC) (National.Research.Council, 2007; Krewski et al., 2010) has led to a resurgence in screening chemicals to rank and prioritize them for further testing. Critical to the success of this approach is the ability to relate the *in vitro* changes to adverse outcomes that are used for regulatory standards settings. Included in the realm of adverse outcomes are altered neurophysiology and changes in integrative functions such as cognitive abilities. Such a linkage can be incorporated into an AOP framework (Figure 1). Within this framework, neurophysiology plays a critical role in defining *functional* changes that can be related to both molecular/biochemical alterations, as well as behavioral changes *in vivo*.

### *In Vitro* Approaches

Several lines of research are already underway to use neurophysiological methods to screen for functional changes produced by toxicants. Examples include higher throughput patch clamp methods (Dunlop et al., 2008; Obergrussberger et al., 2016; Obergrussberger et al., 2018; Liu et al., 2019; Gao et al., 2020), *in vitro* cell culture multi-electrode arrays (MEA) (Mack et al., 2014; Zwartsen et al., 2018; Shafer, 2019), and use of alternative (non-mammalian) species such as zebrafish (Milan et al., 2006; Meyer et al., 2016), or *Caenorhabditis elegans* (*C. elegans*), (Richmond and Jorgensen, 1999; Goodman et al., 2012; Lockery et al., 2012). The use of MEAs has been proposed as a method to screen for seizurogenic potential of chemicals/drugs, and has been used in human tissue for epilepsy studies (Dossi et al., 2014; Meyer et al., 2016; Cho et al., 2017; Bradley and Strock, 2019; Fan et al., 2019). Additionally, MEAs have been proposed to have some utility in classifying possible mechanisms of actions of chemicals on the neuronal activity (Mack et al., 2014). Recently, MEA recordings have been used to provide *in vitro* evidence of altered neurophysiology in dorsal root ganglion cells (DRG) (Johnstone et al., 2020) based on *in silico* predictions (Melnikov et al., 2020). The continued development of the types of neurophysiological methods described above will allow scientists to detect and prioritize chemicals for further testing, based on changes in neuronal function, and to guide more targeted testing strategies with potential mechanistic information.

The future will require advancements using *in vitro* models to generate mechanistic data beyond screening applications. Applications using either human embryonic stem cells or induced pluripotent cells may allow better homology with human responses than rodent cells. Using cell culture conditions which allow development of electrically active three-dimensional (3D) cultures (Dingle et al., 2015; Sandström et al., 2017) may also help recapitulate the human nervous system, and such models have been proposed to study human neurodegenerative diseases (Jorfi et al., 2018; Faravelli et al., 2020). Such 3D models have been reported to have greater development of synaptic and ion transport mechanisms than two-dimensional cultures, suggesting that the neurons are in a higher state of maturation (Simão et al., 2018). In an example using electrical activity, the MEA activity of human neurospheres has been shown to be altered by methyl mercury, in the absence of changes in cell proliferation (Ylä-Outinen et al., 2010). Using a calcium flux measure, chemicals from multiple classes (drugs, flame retardants, industrial chemicals, poly-aromatic hydrocarbons, or pesticides) were tested in neurospheres and ranked for potency (Sirenko et al., 2019). Because this is a developing area of science, it is important to recognize the role and current limitations of electrophysiological measures in brain spheroids and brain-on-chip models (Forro et al., 2021). The technical issues such as planar vs. 3D electrodes, silicon electrodes, mesh electrodes, etc … are largely due to the relative recentness of this area of science. Many of these electrophysiological areas for advancement are similar as those for *in vivo* research (see below). All such *in vitro* models will require studies related to brain-related biology such as regional specific neural differences, hormonal influences on neuronal function, or gender-specific neuronal traits. Regional differences in neuronal composition may be assisted by bioprinting technologies using multiple cell types (see Zhuang et al., 2018 for review). However, the largest challenge will be inclusion of measures for cognitive function or emotion (Fritsche et al., 2018).

Although not restricted to electrophysiological preparations, methods will need to adequately not only model the diversity of neuronal cell types, but additional biological physiological functions will need to be included which can alter toxicokinetics for *in vitro* methods. While all areas of toxicology benefit from accurate modeling of chemical exposure, the blood-brain barrier (BBB) adds an additional level of complexity when considering the nervous system (Gumbleton and Audus, 2001; Bagchi et al., 2019). A developed BBB consists of endothelial cells connected by tight junctions, astrocytic projections, and the extracellular matrix, and can act as a barrier to passage of substances into the brain (Ward and Lamanna, 2004; van Der Helm et al., 2016). Additionally, transporter proteins such a p-glycoprotein can move substances out (or into) the brain (Banks, 1999). Inclusion of these additional cell types and proteins to *in vitro* or *in silico* models (Shityakov and Förster, 2018) will be required to improve dosimetry estimates. Advances in this area have included transwell systems (Stone et al., 2019) co-culture models, and microfluidic approaches using 3D cultures (Bagchi et al., 2019; Choi et al., 2020; Staicu et al., 2021). Such advances will need to be included in toxicity testing to closer reflect the biology of the *in vivo* situation.

### *In Vivo* Neural Assessment

There have been some recent *in vivo* neurophysiological technique developments that allow assessment of both larger neurons and small sensory fibers and may provide some mechanistic insight into changes in peripheral motor or sensory nerve function. In contrast to traditional neurophysiological techniques, these methods use a series of nerve excitability tests that are translatable from humans to animal models. Several of the tests involve electrical conditioning pulses (some are 100 ms or longer) and track the stimulus intensity required to produce a criterion response in the nerve or muscle. The conditioning pulses activate of inactivate various ion channels, allowing some mechanistic interpretation of changes in neuronal responses (Bostock et al., 1998; Nodera and Kaji, 2006; Krishnan et al., 2008; Krishnan et al., 2009). These methods have been translated to both animal models and *in vitro* preparations (Maurer et al., 2007; Boërio et al., 2009; Mori et al., 2010; Nodera and Rutkove, 2012; Arnold et al., 2017), and have been begun to be used in a toxicological context. For example, the acute symptoms of treatment with oxaliplatin have been attributed to slowed inactivation of sodium channels (Heide et al., 2018), and changes in nerve excitability after Wallerian degeneration have been studied (Sawai et al., 2008). Importantly, these neurophysiological methods can be used repeatedly over time, allowing the onset and recovery of toxicological insult to be monitored (Nasu et al., 2014; Sung et al., 2014; Heide et al., 2018). Nerve excitability methods have also been used to assess small sensory fibers (Maurer et al., 2007; Howells et al., 2018), which are technically difficult to test (Bostock et al., 2003; George et al., 2007; Serra et al., 2010). Other investigators have used more traditional histopathological and neurophysiological techniques to assess small nerve fibers, using rectification of multiunit activity and binning the latencies into different ranges, to represent different conduction velocities (correlated with different sizes of nerves) (Zotova et al., 2008; Zotova and Arezzo, 2013). Recently, *in vitro* models for neurophysiological and histological assessment of rodent dorsal root ganglion or induced human motor nerve cells have been reported for assessment of chemotherapy-induced peripheral neuropathy with proposed expansion into environmental chemicals (Sharma et al., 2019; Anderson et al., 2021; Pollard et al., 2021). While these methods are not high throughput, continued development and application of such methods can start to bridge the mechanistic/functional gap in neurophysiological testing of sensory and motor fibers, which are known to be sensitive to toxicants (Le Quesne, 1978; London and Albers, 2007).

### Network Connectivity

Another critical direction for neurophysiological investigations is the interrogation of network connectivity. Neurons, while affected by toxicants individually, function as part of an integrated network. For years, investigators have used *in vitro* approaches such as brain slices as a reductionist approach to study neuronal networks (Dingledine et al., 1980; Joy and Albertson, 1985; Joy and Albertson, 1987; Joy et al., 1989; Gilbert, 2004). Recently, technological advances have allowed patch clamp investigations in brain slices (including human slices) to look at network connectivity (Radnikow et al., 2011; Peng et al., 2019). Other investigators have used cell culture-based *in vitro* MEA to begin to look at formation of synaptic connectivity and network interactions between cells (Jimbo et al., 2003; Berdondini et al., 2006; Müller et al., 2015). Formation of functional networks on MEAs has been described (Erickson et al., 2008; Robinette et al., 2011), and the influence of chemicals to alter network-related endpoints such as coordinated bursting or synchronous firing has been investigated (Brown et al., 2016; Frank et al., 2018). A mechanistic hypothesis for synaptic plasticity in hippocampal neurons cultured on MEAs has been proposed to involve NMDA receptors and ERK1/2 signaling, along with gene transcription and protein synthesis, for maintenance of synchronous bursting for days (Arnold et al., 2005). The need for assessments of neuronal connectivity is critical in a developmental context, and can also be assessed using alternative species such as zebrafish (Miller et al., 2018). To truly assess the impact of chemicals on *in vivo* integrated neuronal function, it is essential to include network interactions as a future direction for both *in vitro* and *in vivo* work.

Assessment of complex neuronal function (such as cognitive or sensory alterations) using neurophysiological techniques will require incorporation of methods developed in basic neuroscience research with continued use and development of methods applicable to humans. As described earlier in this manuscript, neurophysiological methods to assess changes in sensory or cognitive processing in humans have been described (Hansen and Hillyard, 1980; Verleger, 1988; Wijers et al., 1989; Jodo and Kayama, 1992; Vogel and Luck, 2000; Polich, 2007). Assessment of sensory perception is likely to continue to rely on evoked response methods (as described above), with the inclusion of larger arrays of electrodes to allow assessment of changes in the topography of neural responses (Junghöfer et al., 2000; Robinson et al., 2017). Interrogation of complex sensory or cognitive processing will require assessment of networks of neurons and/or brain regions (Buzsáki, 2004; Urai et al., 2021). Neurophysiology is uniquely suited for recording neuronal responses in virtually “real time”, in contrast to the longer time periods usually required for *in vivo* imaging methods (D’Esposito et al., 1999; Sack and Linden, 2003; Trachtenberg et al., 2002). To adequately map neuronal network responses, an array of electrodes is necessary. In humans, these are typically surface electrodes and may be coupled with performance of a behavioral task (Bekker et al., 2004; Sänger et al., 2014). However, mechanistic studies to provide the biological basis for these responses will require animal models and often involve implanted electrodes.

The recording and interpretation of neuronal network activity *in vivo* is an ongoing effort in neuroscience. Advancements in electrode probe materials such as silicon (Senzai et al., 2019; Timme et al., 2021), carbon fiber (Kozai et al., 2012), or mesh probes that can be based on nanotechnology (Liu, 2015; Xie et al., 2015) have allowed long-term recordings of neuronal network responses. Multiple probes, each with multiple electrodes, allow sampling a three-dimensional array of neuronal activity. Multi-electrode methods allow assessment of the timing and correlation of firing between many neurons simultaneously in both anesthetized preparations and in alert and behaving animals. An eight probe array (with 64 recording sites) has been used to examine activity in the rat somatosensory and prefrontal cortex (Barthó et al., 2004). By examining the three-dimensional location of the neurons, the first movement of the autocorellogram, action potential waveform duration, and mean firing rates, the neurons were able to be classified as pyramidal cells or interneurons. A series of probes has been developed with over 5,000 recording sites, with 768 sites recorded simultaneously (Steinmetz et al., 2020) over an eight week period—allowing incorporation into future behavioral paradigms. Using a head-fixed preparation, about 30,000 neurons were recorded from 42 brain regions during multiple sessions of a visual discrimination task in mice. The regional brain responses to ipsilateral vs contralateral choices, and engagement timing were differentiated (Steinmetz et al., 2019). These multi-electrode methods allow assessment of cell outputs (action potentials) with great fidelity. However, it is much more difficult to measure the multiple inputs to a neuron’s dendrites and spines. One approach to looking for changes in cellular inputs can involve measuring the extracellular current flow using a technique known as current source density analysis (CSD) (Mitzdorf, 1985; Szymanski et al., 2009; Senzai et al., 2019). While CSD does not have widespread use in the neurotoxicology field, it has been used to show increased current flow into the supragranular layer of the auditory cortex in rats after an acute dose of salicylate (Stolzberg et al., 2012), indicating changes in intracortical microcircuits. Other investigators have used CSD analysis to show reduction in electrical sinks in the stratum moleculare and decreased distance from the peak inward current (sink) to the granule cell layer of the hippocampus, suggesting a loss of entorhinal afferents to the hippocampal outer molecular layer after 20 weeks of ethanol exposure (Abraham and Hunter, 1982). Therefore, application of multi-electrode analysis, coupled with CSD techniques can begin to assess both neuronal network circuitry and alterations in synaptic inputs (field potentials).

### Cognitive Function

The assessment of changes in cognitive function produced by toxicants is an on-going challenge. Once again, principles and advances in neuroscience will have to be adapted to advance toxicological procedures. Knowing where and when to look for altered physiological responses will remain an important question. Combinations of technologies, such as EEG, magnetoencephalography, and fMRI, may be used to examine sources of cognitive responses (Min et al., 2020). The role of frequency-based assessment of EEG has been linked to communication between different brain regions, memory formation, and other cognitive processes in both human and animal models (Lachaux et al., 2012). In humans. decreased power in alpha and beta EEG frequencies, and reductions in P300 power, were found in high load working memory tasks (Chuang et al., 2019). However, investigations may also continue to rely on evoked responses to interrogate complex brain functions. As some examples, the P3 potential resulting from olfactory stimulation in humans was shown to be modulated by attention in the inferior frontal cortex, insula, and inferior temporal gyrus (Singh et al., 2019). Increased reaction time variability and a reduced amplitude of the P3 potential has been associated with increased ADHD Problems Scale scores on the Child Behavior Checklist (Liu et al., 2020). In rats, peak P2 was found to be related to target detection in an auditory go/no go task, but P3 was not altered (as frequently seen in humans). Increased low frequency power (1–7 Hz) was observed in the frontal cortex on hit trials, but 8–14 Hz power (alpha frequency range) was suppressed, compared to correct rejections (Nanda et al., 2020). These studies illustrate the power of neurophysiological techniques to study cognitive processing in real time, albeit with some differences between rodent models and humans, with an extensive range of cognitive processing remaining to be investigated.

### Perturbation Methodology: Optogenetics

Using methods to perturb normal brain function in animal models will remain an important approach to uncover toxicological mechanisms. Traditional methods such as electrical stimulation, lesions, and pharmacological manipulations can still provide important mechanistic information (Nadler and Cuthbertson, 1980). A well-known caveat to lesion and electrical stimulation methods, is the non-specific nature of the technique. Not only are neuronal cell bodies affected, but so are axons of passage. Pharmacological manipulations can target specific types of neurons/receptors, etc … but may involve a relatively longer time scale of effects and may involve multiple brain regions (Cassaday et al., 1991; Singh et al., 1998; Berman et al., 2002).

Many of these limitations can be overcome with application of the relatively new technique of optogenetics, coupled with neurophysiology. Optogenetic tools allow targeting specific types of neurons with excitatory or inhibitory opsins, allowing neuron function to be controlled with light pulses in real time, and can be integrated with electrophysiology and neuroanatomical methods (Kim et al., 2017; Kuleshova, 2019). Optogenetic inhibition has been used to study the flow of neural network information from sensory to motor areas of the cortex in mice (Guo et al., 2014). Changes in EEG activity have also been examined using optogenetic techniques. For example, modulation of hippocampal theta activity by somatostatin positive, but not parvalbumin positive, GABAergic neurons in a mouse model of Alzheimer’s disease has been demonstrated (Chung et al., 2018). Optogenetic re-activation of dentate gyrus neurons that were first activated during fear conditioning, induced freezing behavior in a different environmental context—indicating that these specific neurons were contributing to the memory engram (Liu et al., 2012). Optogenetic stimulation of auditory presynaptic inputs to the lateral amygdala has been shown to serve as a conditioned stimulus (CS) during fear conditioning, substituting for an auditory stimulus. This study showed the importance of auditory inputs (traditional CS for fear training) to the lateral amygdala in fear memory formation (Kwon et al., 2014). The role of dopaminergic and GABAergic neurons in emotional reward (salience) during motivational behaviors has been dissected using optogenetic methods (Nieh et al., 2013). Closed-loop optogenetic techniques are under development that allow the brain’s neuronal activity to control the optogenetic stimulation of neurons, resulting in extremely naturalistic stimulation paradigms (Grosenick et al., 2015; Bolus et al., 2018). Additionally, optogenetic and neurophysiological techniques can be used in conjunction with other methods such as fMRI, voltage imaging, calcium imaging, and neurotransmitter release (Liang et al., 2015; Renault et al., 2015; Burmeister et al., 2018; Adam et al., 2019) to examine the mechanistic basis of changes produced by xenobiotics on complex brain network functions, such as learning and memory, in nearly real time. Application of these sorts of tools in a toxicological setting can provide valuable mechanistic information that is related to changes in behavior.

## CONCLUSIONS

To adequately solve the problems facing neurotoxicology, the generation of mechanistic data to fill data gaps and allow the construction of AOP networks is needed. Understanding the biological pathways involved in toxicological alterations will enable the predictive validity of *in vitro* screens and the physiological relevance of omic-based changes produced by toxicants to be verified. This type of validity is essential for regulatory purposes and will increase the translational relevance to humans. Given the impetus to use *in vitro* methods as the basis for future risk assessments (National Research Council, 2007; Krewski et al., 2010), the benefit/cost of animal research needs to be considered. At the current time, there is insufficient scientific knowledge to adequately develop *in vitro* tests to adequately assess and protect higher cognitive functions. Thus, judicious and hypothesis-driven *in vivo* research to determine mechanistic key events in AOPs and Integrated Approaches to Testing and Assessments (Organisation for Economic Co-Operation and Development, 2016) will provide toxicological linkages for regulatory purposes and allow the development of batteries of *in vitro* assays to eventually replace the majority of animal testing. It is the change in brain *function* that is of concern to the public. Since the 1700’s, neurophysiology has been uniquely positioned to bridge the gap between mechanistic studies and *in vivo* alterations of the brain’s neuronal networks, helping to fulfill the promise of systems biology to protect human and ecological health.

## Figures and Tables

**TABLE 1 |:** Example types of neuroelectrophysiological methods.

Peripheral/Central Nervous System	Advantages	Considerations
	Can use primary cell culture, immortalized cell lines, iPSC, neurospheres	
Single Electrode	Mechanistic information can include single channel function	Single cell, typically low throughput
Electrode Array	Network level effects, higher throughput (48 and 96 well plates)	Cell type/mechanism impacted can be unclear
**Ex Vivo Methods**		
	Mechanistic information from “more intact” preparation	
Neuromuscular Junction	Isolate changes to pre- vs postsynaptic changes in neuromuscular transmission; Long history of use in multiple species	Isolated from influences of intact central nervous system; Specialized preparation; Human examples are rare
Excised Peripheral Nerve	Action potential conduction velocities; Influences of specific ions can be examined	Limited to single nerve measures; No information on interactions with other nerves
Brain Slice	Known circuitry. Change in long-term potentiation, paired pulse inhibition, kindling; May reflect neuroplasticity	Low throughput; Often more successful in early post-natal animals
**In Vivo Methods**		
**Neuromuscular Recordings**	Human clinical interpretation	Important to control temperature effects
M-Wave	Muscle response following stimulation of motor neurons; Can assess changes in large motor neurons/neuromuscular junction	No assessment of sensory neurons; Must determine nerve vs muscle effects
Distal Latency	Time from stimulation to M-Wave; Assess speed of conduction in motor neurons	No assessment of sensory neurons
F-Wave	Muscle response recorded after antidromic activation of motor neuron; Assess entire length of large motor neurons	Changes may reflect subtle alterations in nerve fiber composition; Difficult to assess changes in motor neuron excitability; Possibly altered by supraspinal/spinal interneuron influences
H-Wave	Muscle response after orthodromic activation of afferents in motor neuron; Includes sensory component; Correlations with sensorymotor neuropathy	Possibly altered by supraspinal/spinal interneuron influences; Not easily measured in all muscles
Repetitive Nerve Stimulation	M-waves recorded after repetitive stimulation of motor neuron; Can identify deficits in presynaptic vs postsynaptic neuromuscular changes	Need to assess neuromuscular units altered by disease/toxicants; Movement artifacts need to be controlled
Single Fiber Electromyography	Record extracellular action potentials from single muscle fibers with repetitive activation; Can detect changes in neuromuscular function (such as jitter) not detected by RNS; Assess safety factor for neuromuscular transmission; Can use stimulation or normal contraction techniques	Requires needle electrodes; Movement artifacts need to be controlled; Should assess multiple neuromuscular junctions
Electromyographic Activity	Can detect changes due to denervation/reinnervation, Active contraction or spontaneous	May require needle electrodes; Typically, only involves superficial muscles; Movement artifacts need to be controlled; Need to identify nerves/muscles affected by toxicity
**Peripheral Nerve**	Human clinical interpretations	Important to control temperature effects
Compound Nerve Action Potential	Ability to detect changes in larger axons, or distribution of axon sizes within a nerve	Difficult to assess small sized axons without specialized techniques
Nerve Conduction Velocity	Measured between two sites on the nerve. Interpretation of changes in myelin or axon size are accepted	Standard methods do not assess small fibers; Testing nonsuperficial nerves can be difficult; Determining exact distance along actual nerve may not be possible
Small Fiber	Can assess changes in small nerve fibers	Can be technically challenging; Not all types of small nerve fibers are assessed
Threshold Tracking	Can assess changes in various ion channel function	Requires specialized equipment/software; Not a large toxicological database
**Peripheral/Central Nervous System**	Human clinical interpretations	Important to control temperature effects
Electroretinogram	Waveforms reflect transmission through photoreceptors, bipolar cells, and ganglion cells for pattern stimulation; Can measure sensory thresholds which are analogous to psychophysical	Requires control of ambient light and light adaptation of subject; May require anesthesia
Somatosensory	Waveforms reflect neurotransmission through lateral or dorsal spinal columns, brainstem dorsal column nuclei (or cerebellum), thalamic nuclei, thalamocortical projections, and neurons in the somatosensory cortex; Can use electrical or “natural” stimuli; Can measure sensory thresholds which are analogous to psychophysical procedures	Primarily assesses large diameter neurons; Usually involves signal averaging
Auditory	Waveforms reflect neurotransmission through auditory nerve, cochlear nucleus, olivary nuclei, lateral lemniscus, inferior colliculus, medial geniculate nucleus, auditory radiation, auditory cortex; Can use pure tones to assess frequency-dependent changes; Can measure sensory thresholds which are analogous to psychophysical procedures	Requires control of auditory stimulus and testing room noise; Usually involves signal averaging
Visual	Waveforms reflect neurotransmission through retinal photoreceptors and ganglion cells, optic nerve and tract, lateral geniculate, thalamocortical projections, visual cortex; Pattern stimuli can allow selectivity for different cell populations; Can measure sensory thresholds which are analogous to psychophysical procedures	Pattern stimulation requires specialized equipment/software; Requires control of lighting conditions during testing; Usually involves signal averaging
Motor Threshold Tracking	Electrical or magnetic stimulation; Assess function of descending motor tracts and peripheral motor nerves	Specialized equipment
Hippocampus	Known circuitry. Change in long-term potentiation, paired pulse inhibition, kindling; Can have mechanistic interpretations, Some tests reflect neuroplasticity	Low throughput; Specialized equipment; Only select human test correlates; Usually animal models
Seizures	Clinical applications; Gold standard for seizurogenic chemicals; Can localize seizurogenic sites	Animal-human extrapolation
Electroencephalography	Clinical applications; Responses can reflect higher cortical processing	Extrapolation of cognitive potentials between animals-humans may be difficult
Single Units	Ability to study network connectivity; Examine specific cell populations; Long history of analyzing brain function; Application of optogenetics	Selectivity bias for larger cells; Must consider animal-human differences; Usually animal models

## References

[R1] Abeyratne UR, Kinouchi Y, Oki H, Okada J, Shichijo F, and Matsumoto K (1991). Artificial Neural Networks for Source Localization in the Human Brain. Brain Topogr 4 (1), 3–21. doi:10.1007/BF01129661 1764347

[R2] Abraham WC, and Hunter BE (1982). An Electrophysiological Analysis of Chronic Ethanol Neurotoxicity in the Dentate Gyrus: Distribution of Entorhinal Afferents. Exp. Brain Res 47 (1), 61–68. doi:10.1007/BF00235887 6288435

[R3] Abraham WC, Jones OD, and Glanzman DL (2019). Is Plasticity of Synapses the Mechanism of Long-Term Memory Storage? Npj Sci. Learn 4 (1), 9. doi:10.1038/s41539-019-0048-y 31285847 PMC6606636

[R4] Adam Y, Kim JJ, Lou S, Zhao Y, Xie ME, Brinks D, (2019). Voltage Imaging and Optogenetics Reveal Behaviour-dependent Changes in Hippocampal Dynamics. Nature 569 (7756), 413–417. doi:10.1038/s41586-019-1166-7 31043747 PMC6613938

[R5] Adrian ED, and Matthews BHC (1934). The Interpretation of Potential Waves in the Cortex. J. Physiol 81 (4), 440–471. doi:10.1113/jphysiol.1934.sp003147 16994555 PMC1394145

[R6] Adrian ED (1926). The Impulses Produced by Sensory Nerve Endings. J. Physiol 61 (1), 49–72. doi:10.1113/jphysiol.1926.sp002273 16993776 PMC1514809

[R7] Adrian ED, and Zotterman Y (1926). The Impulses Produced by Sensory Nerve Endings. J. Physiol 61 (4), 465–483. doi:10.1113/jphysiol.1926.sp002308 16993807 PMC1514868

[R8] Albee RR, Nitschke KD, Mattsson JL, and Stebbins KE (1997). Dichloroacetylene: Effects on the Rat Trigeminal Nerve Somatosensory Evoked Potential. Neurotoxicology and Teratology 19 (1), 27–37. doi:10.1016/s0892-0362(96)00182-1 9088008

[R9] Anderson WA, Bosak A, Hogberg HT, Hartung T, and Moore MJ (2021). Advances in 3D Neuronal Microphysiological Systems: towards a Functional Nervous System on a Chip. In Vitro Cell.Dev.Biol.-Animal 57 (2), 191–206. doi:10.1007/s11626-020-00532-8 PMC7802613 33438114

[R10] Arezzo JC, Litwak MS, and Zotova EG (2011). Correlation and Dissociation of Electrophysiology and Histopathology in the Assessment of Toxic Neuropathy. Toxicol. Pathol 39 (1), 46–51. doi:10.1177/0192623310390231 21119050

[R11] Arnold FJL, Hofmann F, Bengtson CP, Wittmann M, Vanhoutte P, and Bading H (2005). Microelectrode Array Recordings of Cultured Hippocampal Networks Reveal a Simple Model for Transcription and Protein Synthesis-dependent Plasticity. J. Physiol 564 (1), 3–19. doi:10.1113/jphysiol.2004.077446 15618268 PMC1456059

[R12] Arnold R, Moldovan M, Rosberg MR, Krishnan AV, Morris R, and Krarup C (2017). Nerve Excitability in the Rat Forelimb: a Technique to Improve Translational Utility. J. Neurosci. Methods 275, 19–24. doi:10.1016/j.jneumeth.2016.10.013 27771307

[R13] Asadzadeh S, Yousefi Rezaii T, Beheshti S, Delpak A, and Meshgini S (2020). A Systematic Review of EEG Source Localization Techniques and Their Applications on Diagnosis of Brain Abnormalities. J. Neurosci. Methods 339, 108740. doi:10.1016/j.jneumeth.2020.108740 32353472

[R14] Bagchi S, Chhibber T, Lahooti B, Verma A, Borse V, and Jayant RD (2019). *In-vitro* Blood-Brain Barrier Models for Drug Screening and Permeation Studies: an Overview. Dddt 13, 3591–3605. doi:10.2147/dddt.s218708 31695329 PMC6805046

[R15] Baker CL Jr, Hess RR, Olsen BT, and Zrenner E (1988). Current Source Density Analysis of Linear and Non-linear Components of the Primate Electroretinogram. J. Physiol 407 (1), 155–176. doi:10.1113/jphysiol.1988.sp017408 3256615 PMC1191196

[R16] Banks WA (1999). Physiology and Pathology of the Blood-Brain Barrier: Implications for Microbial Pathogenesis, Drug Delivery and Neurodegenerative Disorders. J. Neurovirol 5 (6), 538–555. doi:10.3109/13550289909021284 10602396

[R17] Barthó P, Hirase H, Monconduit L, Zugaro M, Harris KD, and Buzsáki G (2004). Characterization of Neocortical Principal Cells and Interneurons by Network Interactions and Extracellular Features. J. Neurophysiol 92 (1), 600–608. doi:10.1152/jn.01170.2003 15056678

[R18] Beck A (1890). Die Bestimmung der Localisation der Gehirn-und Ruckenmarksfunctionen vermittelst der elektrischen Erscheinungen. Centralblatt fur Physiologie 4, 473–476.

[R19] Bekker EM, Kenemans JL, and Verbaten MN (2004). Electrophysiological Correlates of Attention, Inhibition, Sensitivity and Bias in a Continuous Performance Task. Clin. Neurophysiol 115 (9), 2001–2013. doi:10.1016/j.clinph.2004.04.008 15294202

[R20] Benignus VA, Boyes WK, Hudnell HK, Frey CM, and Svendsgaard DJ (1991). Quantitative Methods for Cross-Species Mapping (CSM). Neurosci. Biobehavioral Rev 15 (1), 165–171. doi:10.1016/s0149-7634(05)80110-1 2052192

[R21] Berdondini L, Chiappalone M, Van Der Wal PD, Imfeld K, de Rooij NF, Koudelka-Hep M, (2006). A Microelectrode Array (MEA) Integrated with Clustering Structures for Investigating *In Vitro* Neurodynamics in Confined Interconnected Sub-populations of Neurons. Sensors Actuators B: Chem 114 (1), 530–541. doi:10.1016/j.snb.2005.04.042

[R22] Berger H (1929). Über das Elektrenkephalogramm des Menschen. Archiv F. Psychiatrie 87 (1), 527–570. doi:10.1007/bf01797193

[R23] Berman FW, LePage KT, and Murray TF (2002). Domoic Acid Neurotoxicity in Cultured Cerebellar Granule Neurons Is Controlled Preferentially by the NMDA Receptor Ca2+ Influx Pathway. Brain Res 924 (1), 20–29. doi:10.1016/S0006-8993(01)03221-8 11743991

[R24] Bernard C (1857). Leçons sur les effets des substances toxiques et médicamenteuses Paris: J.-B. Baillière et fils. 2054698

[R25] Bernstein J (1912). Historisches und Einleitung. Elektrobiologie Braunschweig, Friedr: Vieweg und Sohn, 1–20. doi:10.1007/978-3-663-01627-4_1

[R26] Bernstein J (1868). Ueber den zeitlichen Verlauf der negativen Schwankung des Nervenstroms. Pflüger, Arch 1 (1), 173–207. doi:10.1007/bf01640316

[R27] Bernstein J (1871). Untersuchungen über den Erregungsvorgang im Nerven-und Muskelsysteme Heidelberg: Winter’s Unisersitatsbuchhandlung.

[R28] Bliss TVP, and Lømo T (1973). Long-lasting Potentiation of Synaptic Transmission in the Dentate Area of the Anaesthetized Rabbit Following Stimulation of the Perforant Path. J. Physiol 232 (2), 331–356. doi:10.1113/jphysiol.1973.sp010273 4727084 PMC1350458

[R29] Boërio D, Greensmith L, and Bostock H (2009). Excitability Properties of Motor Axons in the Maturing Mouse. J. Peripher. Nervous Syst 14 (1), 45–53. doi:10.1111/j.1529-8027.2009.00205.x 19335539

[R30] Bolus MF, Willats AA, Whitmire CJ, Rozell CJ, and Stanley GB (2018). Design Strategies for Dynamic Closed-Loop Optogenetic Neurocontrolin Vivo. J. Neural Eng 15 (2), 026011. doi:10.1088/1741-2552/aaa506 29300002 PMC5957547

[R31] Bostock H, Campero M, Serra J, and Ochoa J (2003). Velocity Recovery Cycles of C Fibres Innervating Human Skin. J. Physiol 553 (2), 649–663. doi:10.1113/jphysiol.2003.046342 12963801 PMC2343583

[R32] Bostock H, Cikurel K, and Burke D (1998). Threshold Tracking Techniques in the Study of Human Peripheral Nerve. Muscle Nerve 21 (2), 137–158. doi:10.1002/(sici)1097-4598(199802)21:2<137:aid-mus1>3.0.co;2-c 9466589

[R33] Boyes WK (1994). Rat and Human Sensory Evoked Potentials and the Predictability of Human Neurotoxicity from Rat Data. Neurotoxicology 15 (3), 569–578. 7854590

[R34] Boyes WK, Bercegeay M, Krantz T, Evans M, Benignus V, and Simmons JE (2005). Momentary Brain Concentration of Trichloroethylene Predicts the Effects on Rat Visual Function. Toxicol. Sci 87 (1), 187–196. doi:10.1093/toxsci/kfi242 15976185

[R35] Boyes WK (1992). “Testing Visual System Toxicity Using Evoked Potential Technology,” in The Vulnerable Brain and Environmental Risks. Editors Isaacson RL and Jensen KF (New York: Plenum Press), 193–222. doi:10.1007/978-1-4615-3326-9_9

[R36] Bradley JA, and Strock CJ (2019). Screening for Neurotoxicity with Microelectrode Array. Curr. Protoc. Toxicol 79 (1), e67. doi:10.1002/cptx.67 30575314

[R37] Brazier MAB (1984). Pioneers in the Discovery of Evoked Potentials. Electroencephalography Clin. Neurophysiology/Evoked Potentials Section 59 (1), 2–8. doi:10.1016/0168-5597(84)90015-7 6198161

[R38] Brown JP, Hall D, Frank CL, Wallace K, Mundy WR, and Shafer TJ (2016). Editor’s Highlight: Evaluation of a Microelectrode Array-Based Assay for Neural Network Ontogeny Using Training Set Chemicals. Toxicol. Sci 154 (1), 126–139. doi:10.1093/toxsci/kfw147 27492221

[R39] Burmeister JJ, Pomerleau F, Quintero JE, Huettl P, Ai Y, Jakobsson J, (2018). In Vivo Electrochemical Studies of Optogenetic Control of Glutamate Signaling Measured Using Enzyme-Based Ceramic Microelectrode Arrays, Biochemical Approaches for Glutamatergic Neurotransmission New York, NY: Humana Press, 327–351. doi:10.1007/978-1-4939-7228-9_11

[R40] Buzsáki G (2004). Large-scale Recording of Neuronal Ensembles. Nat. Neurosci 7 (5), 446–451. doi:10.1038/nn1233 15114356

[R41] Caccia MR, Salvaggio A, Dezuanni E, Osio M, Bevilacqua M, Norbiato G, (1993). An Electrophysiological Method to Assess the Distribution of the Sensory Propagation Velocity of the Digital Nerve in normal and Diabetic Subjects. Electroencephalography Clin. Neurophysiology/Evoked Potentials Section 89 (2), 88–94. doi:10.1016/0168-5597(93)90089-8 7683606

[R42] Carter CS, Braver TS, Barch DM, Botvinick MM, Noll D, and Cohen JD (1998). Anterior Cingulate Cortex, Error Detection, and the Online Monitoring of Performance. Science 280 (5364), 747–749. doi:10.1126/science.280.5364.747 9563953

[R43] Cassaday H, Hodges H, and Gray J (1991). “The Effects of Pharmacological and Neurotoxic Manipulation of Serotonergic Activity on Latent Inhibition in the Rat: Implications for the Neural Basis of Acute Schizophrenia,” in Serotonin-related Psychiatric Syndrome: Clinical and Therapeutic Links. International Congress and Symposium Series Editors Cassano G and Akiskal H (Royal society of Medical Services Limited), 99–105.

[R44] Caton R (1877). Interim Report on Investigation of the Electric Currents of the Brain. Br. Med. J 1, 62.

[R45] Caton R (1875). The Electric Currents of the Brain. Br. Med. J 2, 278.

[R46] Cavaletti G, Tredici G, Braga M, and Tazzari S (1995). Experimental Peripheral Neuropathy Induced in Adult Rats by Repeated Intraperitoneal Administration of Taxol. Exp. Neurol 133 (1), 64–72. doi:10.1006/exnr.1995.1008 7601264

[R47] Cho S-J, Byun D, Nam T-S, Choi S-Y, Lee B-G, Kim M-K, (2017). A 3D-Printed Sensor for Monitoring Biosignals in Small Animals. J. Healthc. Eng 2017 (1), 1–6. doi:10.1155/2017/9053764 PMC5676486 29209491

[R48] Choi J-H, Santhosh M, and Choi J-W (2020). *In Vitro* blood–brain Barrier-Integrated Neurological Disorder Models Using a Microfluidic Device. Micromachines 11 (1), 21. doi:10.3390/mi11100905 PMC7019695 31878184

[R49] Chuang K-Y, Chen Y-H, Balachandran P, Liang W-K, and Juan C-H (2019). Revealing the Electrophysiological Correlates of Working Memory-Load Effects in Symmetry Span Task with HHT Method. Front. Psychol 10, 855. doi:10.3389/fpsyg.2019.00855 31105617 PMC6499155

[R50] Chung H, Park K, Jang HJ, Kohl MM, and Kwag J, 2018. Optogenetic Activation of SST-Positive Interneurons Restores Hippocampal Theta Oscillation Impairment Induced by Soluble Amyloid Beta Oligomers *In Vivo*. bioRxiv, 465112.

[R51] Cliffer KD, Siuciak JA, Carson SR, Radley HE, Park JS, Lewis DR, (1998a). Physiological Characterization of Taxol-Induced Large-Fiber Sensory Neuropathy in the Rat. Ann. Neurol 43 (1), 46–55. doi:10.1002/ana.410430111 9450768

[R52] Cliffer KD, Tonra JR, Carson SR, Radley HE, Cavnor C, Lindsay RM, (1998b). Consistent Repeated M- and H-Wave Recording in the Hind Limb of Rats. Muscle Nerve 21 (11), 1405–1413. doi:10.1002/(sici)1097-4598(199811)21:11<1405:aid-mus7>3.0.co;2-d 9771663

[R53] Coenen A, and Zayachkivska O (2013). Adolf Beck: A pioneer in Electroencephalography in between Richard. Adv. Cogn. Psychol 9, 216–221. doi:10.2478/v10053-008-0148-3 24605179 PMC3902832

[R54] Cole KS (1949). Dynamic Electrical Characteristics of the Squid Axon Membrane. Arch. des Sci. Physiologiques 3 (2), 253–258.

[R55] Collura TF (1993). History and Evolution of Electroencephalographic Instruments and Techniques. J. Clin. Neurophysiol 10 (4), 476–504. doi:10.1097/00004691-199310000-00007 8308144

[R56] Cui S, Duan L, Gong B, Qiao Y, Xu F, Chen J, (2019). EEG Source Localization Using Spatio-Temporal Neural Network. China Commun 16 (7), 131–143. doi:10.23919/jcc.2019.07.011

[R57] D’Esposito M, Zarahn E, and Aguirre GK (1999). Event-related Functional MRI: Implications for Cognitive Psychology. Psychol. Bull 125 (1), 155–164. doi:10.1037/0033-2909.125.1.155 9990848

[R58] Daube JR, and Rubin DI (2009). Needle Electromyography. Muscle Nerve 39 (2), 244–270. doi:10.1002/mus.21180 19145648

[R59] De Koning P, Neijt JP, Jennekens FGI, and Gispen WH (1987). Evaluation of Cis-Diamminedichloroplatinum (II) (Cisplatin) Neurotoxicity in Rats. Toxicol. Appl. Pharmacol 89 (1), 81–87. doi:10.1016/0041-008x(87)90178-5 3590191

[R60] DeJesus CPV, Towfighi J, and Snyder DR (1978). Sural Nerve Conduction Study in the Rat: a New Technique for Studying Experimental Neuropathies. Muscle Nerve 1 (2), 162–167. doi:10.1002/mus.880010210 220531

[R61] Dietsch G (1932). Fourier-analyse von elektrencephalogrammen des menschen. Pflügers Arch 230 (1), 106–112. doi:10.1007/bf01751972

[R62] Dingle Y-TL, Boutin ME, Chirila AM, Livi LL, Labriola NR, Jakubek LM, (2015). Three-Dimensional Neural Spheroid Culture: AnIn VitroModel for Cortical Studies. Tissue Eng. C: Methods 21 (12), 1274–1283. doi:10.1089/ten.TEC.2015.0135 PMC4663656 26414693

[R63] Dingledine R, Dodd J, and Kelly JS (1980). The *In Vitro* Brain Slice as a Useful Neurophysiological Preparation for Intracellular Recording. J. Neurosci. Methods 2 (4), 323–362. doi:10.1016/0165-0270(80)90002-3 6106092

[R64] Dongren Y, Tao L, and Fengsheng H (1999). Electroneurophysiological Studies in Rats of Acute Dimethoate Poisoning. Toxicol. Lett 107 (1–3), 249–254. doi:10.1016/s0378-4274(99)00054-5 10414803

[R65] Dorfman LJ (1984). The Distribution of Conduction Velocities (DCV) in Peripheral Nerves: a Review. Muscle Nerve 7 (1), 2–11. doi:10.1002/mus.880070103 6366539

[R66] Dossi E, Blauwblomme T, Nabbout R, Huberfeld G, and Rouach N (2014). Multi-electrode Array Recordings of Human Epileptic Postoperative Cortical Tissue. JoVE 92 (92), e51870. doi:10.3791/51870 PMC4353385 25407747

[R67] Douglas RM, and Goddard GV (1975). Long-term Potentiation of the Perforant Path-Granule Cell Synapse in the Rat hippocampus. Brain Res 86 (2), 205–215. doi:10.1016/0006-8993(75)90697-6 163667

[R68] du Bois-Reymond E (1848). Untersuchungen Uber Thierische Elektricitat, 1. Berlin, Reimer, 1–816.

[R69] Dunlop J, Bowlby M, Peri R, Vasilyev D, and Arias R (2008). High-throughput Electrophysiology: an Emerging Paradigm for Ion-Channel Screening and Physiology. Nat. Rev. Drug Discov 7 (4), 358–368. doi:10.1038/nrd2552 18356919

[R70] Dyer R (1985). The Use of Sensory Evoked Potentials in Toxicology*1, *2. Fundam. Appl. Toxicol 5 (1), 24–40. doi:10.1016/0272-0590(85)90048-X 3886466

[R71] Eells JT, Henry MM, Lewandowski MF, Seme MT, and Murray TG (2000). Development and Characterization of a Rodent Model of Methanol-Induced Retinal and Optic Nerve Toxicity. Neurotoxicology 21 (3), 321–330. 10894122

[R72] Eells JT, Salzman MM, Lewandowski MF, and Murray TG (1996). Formate-induced Alterations in Retinal Function in Methanol-Intoxicated Rats. Toxicol. Appl. Pharmacol 140 (1), 58–69. doi:10.1006/taap.1996.0197 8806870

[R73] Erickson J, Tooker A, Tai Y-C, and Pine J (2008). Caged Neuron MEA: A System for Long-Term Investigation of Cultured Neural Network Connectivity. J. Neurosci. Methods 175 (1), 1–16. doi:10.1016/j.jneumeth.2008.07.023 18775453 PMC2585802

[R74] Falkenstein M, Hohnsbein J, Hoormann J, and Blanke L (1991). Effects of Crossmodal Divided Attention on Late ERP Components. II. Error Processing in Choice Reaction Tasks. Electroencephalography Clin. Neurophysiol 78 (6), 447–455. doi:10.1016/0013-4694(91)90062-9 1712280

[R75] Fan J, Thalody G, Kwagh J, Burnett E, Shi H, Lewen G, (2019). Assessing Seizure Liability Using Multi-Electrode Arrays (MEA). Toxicol. Vitro 55, 93–100. doi:10.1016/j.tiv.2018.12.001 30528373

[R76] Faravelli I, Costamagna G, Tamanini S, and Corti S (2020). Back to the Origins: Human Brain Organoids to Investigate Neurodegeneration. Brain Res 1727, 146561. doi:10.1016/j.brainres.2019.146561 31758922

[R77] Fechter LD, Gearhart C, Fulton S, Campbell J, Fisher J, Na K, (2007). JP-8 Jet Fuel Can Promote Auditory Impairment Resulting from Subsequent Noise Exposure in Rats. Toxicol. Sci 98 (2), 510–525. doi:10.1093/toxsci/kfm101 17483120

[R78] Finley DB, Wang X, Graff JE, and Herr DW (2009). Single Fiber Electromyographic Jitter and Detection of Acute Changes in Neuromuscular Function in Young and Adult Rats. J. Pharmacol. Toxicol. Methods 59 (2), 108–119. doi:10.1016/j.vascn.2009.02.001 19367692

[R79] Forro C, Caron D, Angotzi G, Gallo V, Berdondini L, Santoro F, (2021). Electrophysiology Read-Out Tools for Brain-On-Chip Biotechnology. Micromachines 12 (2), 124. doi:10.3390/mi12020124 33498905 PMC7912435

[R80] Fox DA, and Rubinstein SD (1989). Age-related Changes in Retinal Sensitivity, Rhodopsin Content and Rod Outer Segment Length in Hooded Rats Following Low-Level lead Exposure during Development. Exp. Eye Res 48 (2), 237–249. doi:10.1016/s0014-4835(89)80073-9 2924811

[R81] Frank CL, Brown JP, Wallace K, Wambaugh JF, Shah I, and Shafer TJ (2018). Defining Toxicological Tipping Points in Neuronal Network Development. Toxicol. Appl. Pharmacol 354, 81–93. doi:10.1016/j.taap.2018.01.017 29397954

[R82] Fritsche E, Barenys M, Klose J, Masjosthusmann S, Nimtz L, Schmuck M, (2018). Current Availability of Stem Cell-Based *In Vitro* Methods for Developmental Neurotoxicity (DNT) Testing. Toxicol. Sci 165 (1), 21–30. doi:10.1093/toxsci/kfy178 29982830

[R83] Gagnaire F, and Marignac B (1999). Electrophysiological Deficiency in Peripheral Nerve Induced by Treatment for 12 Weeks with 2-Butenenitrile, 3-Butenenitrile, Cis-2-Pentenenitrile and 3, 3′-Iminodipropionitrile in Rats. Pharmacol. Toxicol 84 (6), 247–254. doi:10.1111/j.1600-0773.1999.tb01490.x 10401725

[R84] Galvani L (1791). De Viribus Electricitatis in Motu Musculari. Commentarius. De Bonoiensi Scientiarum Artium Intituo Atque Academie Commentarii 7, 363–418.

[R85] Gao J, Zhang H, Xiong P, Yan X, Liao C, and Jiang G (2020). Application of Electrophysiological Technique in Toxicological Study: from Manual to Automated Patch-Clamp Recording. Trac Trends Anal. Chem 133, 116082. doi:10.1016/j.trac.2020.116082

[R86] Gasser HS, and Erlanger J (1927). The Rôle Played by the Sizes of the Constituent Fibers of a Nerve Trunk in Determining the Form of its Action Potential Wave. Am. J. Physiology-Legacy Content 80 (3), 522–547. doi:10.1152/ajplegacy.1927.80.3.522

[R87] Gehring WJ, Goss B, Coles MGH, Meyer DE, and Donchin E (1993). A Neural System for Error Detection and Compensation. Psychol. Sci 4 (6), 385–390. doi:10.1111/j.1467-9280.1993.tb00586.x

[R88] George A, Serra J, Navarro X, and Bostock H (2007). Velocity Recovery Cycles of Single C Fibres Innervating Rat Skin. J. Physiol 578 (1), 213–232. doi:10.1113/jphysiol.2006.116129 17023508 PMC2075106

[R89] Gilbert ME, Mack CM, and Crofton KM (1989). Pyrethroids and Enhanced Inhibition in the hippocampus of the Rat. Brain Res 477 (1–2), 314–321. doi:10.1016/0006-8993(89)91420-0 2702491

[R90] Gilbert ME (1992). A Characterization of Chemical Kindling with the Pesticide Endosulfan. Neurotoxicology and Teratology 14 (2), 151–158. doi:10.1016/0892-0362(92)90063-g 1593989

[R91] Gilbert ME (2004). Alterations in Synaptic Transmission and Plasticity in Area CA1 of Adult hippocampus Following Developmental Hypothyroidism. Developmental Brain Res 148 (1), 11–18. doi:10.1016/j.devbrainres.2003.09.018 14757514

[R92] Gilbert ME, and Mack CM (1998). Chronic lead Exposure Accelerates Decay of Long-Term Potentiation in Rat Dentate Gyrus *In Vivo*. Brain Res 789 (1), 139–149. doi:10.1016/s0006-8993(97)01517-5 9602098

[R93] Gilbert ME, and Mack CM (1995). Seizure Thresholds in Kindled Animals Are Reduced by the Pesticides Lindane and Endosulfan. Neurotoxicology and teratology 17 (2), 143–150. doi:10.1016/0892-0362(94)00065-l 7539098

[R94] Gilbert ME (1995). Repeated Exposure to Lindane Leads to Behavioral Sensitization and Facilitates Electrical Kindling. Neurotoxicology and Teratology 17 (2), 131–141. doi:10.1016/0892-0362(94)00064-k 7539097

[R95] Goddard GV, McIntyre DC, and Leech CK (1969). A Permanent Change in Brain Function Resulting from Daily Electrical Stimulation. Exp. Neurol 25 (3), 295–330. doi:10.1016/0014-4886(69)90128-9 4981856

[R96] Goodman MB, Lindsay TH, Lockery SR, and Richmond JE (2012). Electrophysiological Methods for *Caenorhabditis elegans* Neurobiology. Methods Cel Biol 107, 409–436. doi:10.1016/B978-0-12-394620-1.00014-X PMC3959639 22226532

[R97] Gray TC (1947). The Use of D-Tubocurarine Chloride in Anaesthesia. Ann. R. Coll. Surg. Engl 1 (4), 191–203. 20267902

[R98] Grech R, Cassar T, Muscat J, Camilleri KP, Fabri SG, Zervakis M, (2008). Review on Solving the Inverse Problem in EEG Source Analysis. J. Neuroengineering Rehabil 5 (1), 1–33. doi:10.1186/1743-0003-5-25 PMC2605581 18990257

[R99] Grosenick L, Marshel JH, and Deisseroth K (2015). Closed-loop and Activity-Guided Optogenetic Control. Neuron 86 (1), 106–139. doi:10.1016/j.neuron.2015.03.034 25856490 PMC4775736

[R100] Gumbleton M, and Audus KL (2001). Progress and Limitations in the Use of *In Vitro* Cell Cultures to Serve as a Permeability Screen for the Blood-Brain Barrier. J. Pharm. Sci 90 (11), 1681–1698. doi:10.1002/jps.1119 11745727

[R101] Guo ZV, Li N, Huber D, Ophir E, Gutnisky D, Ting JT, (2014). Flow of Cortical Activity Underlying a Tactile Decision in Mice. Neuron 81 (1), 179–194. doi:10.1016/j.neuron.2013.10.020 24361077 PMC3984938

[R102] Haider M, Spong P, and Lindsley DB (1964). Attention, Vigilance, and Cortical Evoked-Potentials in Humans. Science 145 (3628), 180–182. doi:10.1126/science.145.3628.180 14171563

[R103] Hamers F, van der Hoop RG, Steerenburg PA, Neijt JP, and Gispen WH (1991). Putative Neurotrophic Factors in the protection of Cisplatin-Induced Peripheral Neuropathy in Rats. Toxicol. Appl. Pharmacol 111 (3), 514–522. doi:10.1016/0041-008x(91)90255-d 1660632

[R104] Hansen JC, and Hillyard SA (1980). Endogenous Brain Potentials Associated with Selective Auditory Attention. Electroencephalogr Clin. Neurophysiol 49 (3–4), 277–290. doi:10.1016/0013-4694(80)90222-9 6158404

[R105] Heide R, Bostock H, Ventzel L, Grafe P, Bergmans J, Fuglsang-Frederiksen A, (2018). Axonal Excitability Changes and Acute Symptoms of Oxaliplatin Treatment: *In Vivo* Evidence for Slowed Sodium Channel Inactivation. Clin. Neurophysiol 129 (3), 694–706. doi:10.1016/j.clinph.2017.11.015 29233604

[R106] Helmholtz H (1852). Messungen über fortpflanzungsgeschwindigkeit der reizung in den nerven-zweite reihe. Arch. Anat. Physiol. Wiss Med, 199–216.

[R107] Helmholtz H (1850a). Note sur la vitesse de propagation de l’agent nerveux dans les nerfs rachidiens. CR Acadademy Sci. (Paris) 30, 204–206.

[R108] Helmholtz H (1850b). Über die Fortpflanzungsgeschwindigkeit der Nervenreizung [On the speed of nerve conduction]. Archiv für Anatomie, Physiologie und wissenschaftliche Medicin, 71–73.

[R109] Hermann L (1872). Ueber eine Wirkung galvanischer Ströme auf Muskeln und Nerven. Pflüger, Arch 5 (1), 223–275. doi:10.1007/bf01675805

[R110] Hermann L (1873). Weitere Untersuchungen über den Electrotonus, insbesondere über die Erstreckung desselben auf die intramusculären Nervenenden. Pflüger, Arch 7 (1), 301–322. doi:10.1007/bf01613330

[R111] Herr D, Boyes WK, and Dyer RS (1992). Alterations in Rat Flash and Pattern Reversal Evoked Potentials after Acute or Repeated Administration of Carbon Disulfide (CS2)*1, *2. Fundam. Appl. Toxicol 18 (3), 328–342. doi:10.1016/0272-0590(92)90131-z 1597259

[R112] Herr D, King D, Barone S Jr., and Crofton KM (1995). Alterations in Flash Evoked Potentials (FEPs) in Rats Produced by 3,3′-iminodipropionitrile (IDPN),. Neurotoxicology and Teratology 17 (6), 645–656. doi:10.1016/0892-0362(95)02007-1 8747746

[R113] Herr DW, Vo KT, Morgan DL, and Sills RC (1998). Carbon Disulfide Neurotoxicity in Rats: VI. Electrophysiological Examination of Caudal Tail Nerve Compound Action Potentials and Nerve Conduction Velocity. Neurotoxicology 19 (1), 129–146. 9498229

[R114] Herr DW, and Boyes WK (1995). “Electrophysiological Analysis of Complex Brain Systems,” in Neurotoxicology. Approaches and Methods Editors Chang LWand Slikker W Jr. (New York: Academic Press), 205–221. doi:10.1016/b978-012168055-8/50013-3

[R115] Herr DW, Graff JE, Moser VC, Crofton KM, Little PB, Morgan DL, (2007). Inhalational Exposure to Carbonyl Sulfide Produces Altered Brainstem Auditory and Somatosensory-Evoked Potentials in Fischer 344N Rats. Toxicol. Sci 95 (1), 118–135. doi:10.1093/toxsci/kfl146 17079700

[R116] Heynen H, and Van Norren D (1985). Origin of the Electroretinogram in the Intact Macaque Eye-II. Vis. Res 25 (5), 709–715. doi:10.1016/0042-6989(85)90177-4 4024471

[R117] Hitzig E, and Fritsch G (1870). Über die elektrische Erregbarkeit des Grosshirns. Arch. Anat. Physiol, 300–332.

[R118] Hnik P, Vejsada R, and Kasicki S (1982). EMG Changes in Rat Hind Limb Muscles Following Bilateral Deafferentation. Pflugers Arch 395 (3), 182–185. doi:10.1007/BF00584806 6891455

[R119] Hodgkin AL, and Huxley AF (1952). A Quantitative Description of Membrane Current and its Application to Conduction and Excitation in Nerve. J. Physiol 117 (4), 500–544. doi:10.1113/jphysiol.1952.sp004764 12991237 PMC1392413

[R120] Hodgkin AL, Huxley AF, and Katz B (1952). Measurement of Current-voltage Relations in the Membrane of the Giant Axon of Loligo. J. Physiol 116 (4), 424–448. doi:10.1113/jphysiol.1952.sp004716 14946712 PMC1392219

[R121] Hodgkin AL, and Katz B (1949). The Effect of Sodium Ions on the Electrical Activity of the Giant Axon of the Squid. J. Physiol 108 (1), 37–77. doi:10.1113/jphysiol.1949.sp004310 18128147 PMC1392331

[R122] Hoechstetter K, Bornfleth H, Weckesser D, Ille N, Berg P, and Scherg M (2003). BESA Source Coherence: a New Method to Study Cortical Oscillatory Coupling. Brain Topogr 16 (4), 233–238. doi:10.1023/b:brat.0000032857.55223.5d 15379219

[R123] Hölscher C (1997). Long-term Potentiation: a Good Model for Learning and Memory? Prog. Neuro-Psychopharmacology Biol. Psychiatry 21 (1), 47–68. doi:10.1016/s0278-5846(96)00159-5 9075258

[R124] Howard JF (2013). Electrodiagnosis of Disorders of Neuromuscular Transmission. Phys. Med. Rehabil. Clin. North America 24 (1), 169–192. doi:10.1016/j.pmr.2012.08.013 23177038

[R125] Howd RA, Rebert CS, Dickinson J, and Pryor GT (1983). A Comparison of the Rates of Development of Functional Hexane Neuropathy in Weanling and Young Adult Rats. Neurobehav Toxicol. Teratol 5 (1), 63–68. 6304548

[R126] Howells J, Bostock H, Park SB, Kiernan MC, and Burke D (2018). Tracking Small Sensory Nerve Action Potentials in Human Axonal Excitability Studies. J. Neurosci. Methods 298, 45–53. doi:10.1016/j.jneumeth.2018.02.003 29444448

[R127] Hudnell HK, Boyes WK, and Otto DA (1990). Rat and Human Visual-Evoked Potentials Recorded under Comparable Conditions: a Preliminary Analysis to Address the Issue of Predicting Human Neurotoxic Effects from Rat Data. Neurotoxicology and Teratology 12 (4), 391–398. doi:10.1016/0892-0362(90)90059-l 2392099

[R128] Janssen R, Boyes WK, and Dyer RS (1983). Effects of Chlordimeform on the Brainstem Auditory Evoked Response in Rats. Dev. Toxicol. Environ. Sci 11, 533–536. 6677500

[R129] Janssen R, Schweitzer L, and Jensen KF (1991). Glutamate Neurotoxicity in the Developing Rat Cochlea: Physiological and Morphological Approaches. Brain Res 552 (2), 255–264. doi:10.1016/0006-8993(91)90090-i 1680530

[R130] Jasper HH, Kershman J, and Elvidge A (1940). Electroencephalographic Studies of Injury to the Head. Arch. Neurpsych 44 (2), 328–350. doi:10.1001/archneurpsyc.1940.02280080088005

[R131] Jimbo Y, Kasai N, Torimitsu K, Tateno T, and Robinson HPC (2003). A System for MEA-Based Multisite Stimulation. IEEE Trans. Biomed. Eng 50 (2), 241–248. doi:10.1109/TBME.2002.805470 12665038

[R132] Jodo E, and Kayama Y (1992). Relation of a Negative ERP Component to Response Inhibition in a Go/No-Go Task. Electroencephalography Clin. Neurophysiol 82 (6), 477–482. doi:10.1016/0013-4694(92)90054-l 1375556

[R133] Johnstone AFM, Mack CM, Valdez MC, Shafer TJ, LoPachin RM, Herr DW, (2020). Acute *In Vitro* Effects on Embryonic Rat Dorsal Root Ganglion (DRG) Cultures by In Silico Predicted Neurotoxic Chemicals: Evaluations on Cytotoxicity, Neurite Length, and Neurophysiology. Toxicol. Vitro 69, 104989. doi:10.1016/j.tiv.2020.104989 PMC8056874 32882341

[R134] Jones RD, Hamilton BF, and Dass PD (1995). The Effects of Physostigmine on the Electroretinogram in the Beagle Dog. Vet. Res. Commun 19 (2), 135–147. doi:10.1007/BF01839280 7645197

[R135] Jorfi M, D’Avanzo C, Kim DY, and Irimia D (2018). Three-Dimensional Models of the Human Brain Development and Diseases. Adv. Healthc. Mater 7 (1), 1700723. doi:10.1002/adhm.201700723 PMC5762251 28845922

[R136] Joy RM, and Albertson TE (1985). Effects of Lindane on Excitation and Inhibition Evoked in Dentate Gyrus by Perforant Path Stimulation. Neurobehav Toxicol. Teratol 7 (1), 1–8. 2582285

[R137] Joy RM, and Albertson TE (1987). Interactions of Lindane with Synaptically Mediated Inhibition and Facilitation in the Dentate Gyrus. Neurotoxicology 8 (4), 529–542. 2450321

[R138] Joy RM, Stark LG, and Albertson TE (1982). Proconvulsant Effects of Lindane: Enhancement of Amygdaloid Kindling in the Rat. Neurobehav Toxicol. Teratol 4 (3), 347–354. 6178984

[R139] Joy RM, Stark LG, Peterson SL, Bowyer JF, and Albertson TE (1980). The Kindled Seizure: Production of and Modification by Dieldrin in Rats. Neurobehav Toxicol 2 (2), 117–124. 7290307

[R140] Joy RM, Walby WF, Stark LG, and Albertson TE (1995). Lindane Blocks GABAA-Mediated Inhibition and Modulates Pyramidal Cell Excitability in the Rat Hippocampal Slice. Neurotoxicology 16 (2), 217–228. 7566682

[R141] Joy RM, Albertson TE, and Ray DE (1989). Type I and Type II Pyrethroids Increase Inhibition in the Hippocampal Dentate Gyrus of the Rat. Toxicol. Appl. Pharmacol 98 (3), 398–412. doi:10.1016/0041-008x(89)90169-5 2718171

[R142] Junghöfer M, Elbert T, Tucker DM, and Rockstroh B (2000). Statistical Control of Artifacts in Dense Array EEG/MEG Studies. Psychophysiology 37 (4), 523–532. 10934911

[R143] Kelly SS, Mutch E, Williams FM, and Blain PG (1994). Electrophysiological and Biochemical Effects Following Single Doses of Organophosphates in the Mouse. Arch. Toxicol 68 (7), 459–466. doi:10.1007/s002040050097 7979963

[R144] Kim CK, Adhikari A, and Deisseroth K (2017). Integration of Optogenetics with Complementary Methodologies in Systems Neuroscience. Nat. Rev. Neurosci 18 (4), 222–235. doi:10.1038/nrn.2017.15 28303019 PMC5708544

[R145] Kimura J (1984). Principles and Pitfalls of Nerve Conduction Studies. Ann. Neurol 16 (4), 415–429. doi:10.1002/ana.410160402 6093680

[R146] Kober TE, and Cooper GP (1976). Lead Competitively Inhibits Calcium-dependent Synaptic Transmission in the Bullfrog Sympathetic Ganglion. Nature 262 (5570), 704–705. doi:10.1038/262704a0 183141

[R147] Koles ZJ (1998). Trends in EEG Source Localization. Electroencephalography Clin. Neurophysiol 106 (2), 127–137. doi:10.1016/s0013-4694(97)00115-6 9741773

[R148] Kozai TDY, Langhals NB, Patel PR, Deng X, Zhang H, Smith KL, (2012). Ultrasmall Implantable Composite Microelectrodes with Bioactive Surfaces for Chronic Neural Interfaces. Nat. Mater 11 (12), 1065–1073. doi:10.1038/nmat3468 23142839 PMC3524530

[R149] Krewski D, Acosta D Jr, Andersen M, Anderson H, Bailar JC III, Boekelheide K, (2010). Toxicity Testing in the 21st century: a Vision and a Strategy. J. Toxicol. Environ. Health B 13 (2–4), 51–138. doi:10.1080/10937404.2010.483176 PMC4410863 20574894

[R150] Krishnan AV, Lin CS-Y, Park SB, and Kiernan MC (2008). Assessment of Nerve Excitability in Toxic and Metabolic Neuropathies. J. Peripher. Nerv Syst 13 (1), 7–26. doi:10.1111/j.1529-8027.2008.00155.x 18346228

[R151] Krishnan AV, Lin CS-Y, Park SB, and Kiernan MC (2009). Axonal Ion Channels from Bench to Bedside: a Translational Neuroscience Perspective. Prog. Neurobiol 89 (3), 288–313. doi:10.1016/j.pneurobio.2009.08.002 19699774

[R152] Kuleshova EP (2019). Optogenetics - New Potentials for Electrophysiology. Neurosci. Behav. Physi 49 (2), 169–177. doi:10.1007/s11055-019-00711-5

[R153] Kwon J-T, Nakajima R, Kim H-S, Jeong Y, Augustine GJ, and Han J-H (2014). Optogenetic Activation of Presynaptic Inputs in Lateral Amygdala Forms Associative Fear Memory. Learn. Mem 21 (11), 627–633. doi:10.1101/lm.035816.114 25322798 PMC4201812

[R154] Lachaux J-P, Axmacher N, Mormann F, Halgren E, and Crone NE (2012). High-frequency Neural Activity and Human Cognition: Past, Present and Possible Future of Intracranial EEG Research. Prog. Neurobiol 98 (3), 279–301. doi:10.1016/j.pneurobio.2012.06.008 22750156 PMC3980670

[R155] Le Quesne PM (1978). Clinical Expression of Neurotoxic Injury and Diagnostic Use of Electromyography. Environ. Health Perspect 26, 89–95. doi:10.1289/ehp.782689 363422 PMC1637261

[R156] Leandri M, Ghignotti M, Emionite L, Leandri S, and Cilli M (2012). Electrophysiological Features of the Mouse Tail Nerves and Their Changes in Chemotherapy Induced Peripheral Neuropathy (CIPN). J. Neurosci. Methods 209 (2), 403–409. doi:10.1016/j.jneumeth.2012.07.005 22800858

[R157] Liang Z, Watson GDR, Alloway KD, Lee G, Neuberger T, and Zhang N (2015). Mapping the Functional Network of Medial Prefrontal Cortex by Combining Optogenetics and fMRI in Awake Rats. Neuroimage 117, 114–123. doi:10.1016/j.neuroimage.2015.05.036 26002727 PMC4512884

[R158] Lilienthal H, Heikkinen P, Andersson PL, van der Ven LTM, and Viluksela M (2011). Auditory Effects of Developmental Exposure to Purity-Controlled Polychlorinated Biphenyls (PCB52 and PCB180) in Rats. Toxicol. Sci 122 (1), 100–111. doi:10.1093/toxsci/kfr077 21464466

[R159] Liu C, Li T, and Chen J (2019). Role of High-Throughput Electrophysiology in Drug Discovery. Curr. Protoc. Pharmacol 87 (1), e69. doi:10.1002/cpph.69 31805608

[R160] Liu J, Fu T-M, Cheng Z, Hong G, Zhou T, Jin L, (2015). Syringe-injectable Electronics. Nat. Nanotech 10 (7), 629–636. doi:10.1038/nnano.2015.115 PMC4591029 26053995

[R161] Liu X, Ramirez S, Pang PT, Puryear CB, Govindarajan A, Deisseroth K, (2012). Optogenetic Stimulation of a Hippocampal Engram Activates Fear Memory Recall. Nature 484 (7394), 381–385. doi:10.1038/nature11028 22441246 PMC3331914

[R162] Liu Y, Hanna GL, Hanna BS, Rough HE, Arnold PD, and Gehring WJ (2020). Behavioral and Electrophysiological Correlates of Performance Monitoring and Development in Children and Adolescents with Attention-Deficit/hyperactivity Disorder. Brain Sci 10 (2), 79. doi:10.3390/brainsci10020079 PMC7071615 32024242

[R163] Lockery SR, Hulme SE, Roberts WM, Robinson KJ, Laromaine A, Lindsay TH, (2012). A Microfluidic Device for Whole-Animal Drug Screening Using Electrophysiological Measures in the Nematode *C. elegans*. Lab. Chip 12 (12), 2211–2220. doi:10.1039/c2lc00001f 22588281 PMC3372093

[R164] Lømo T (1971). Patterns of Activation in a Monosynaptic Cortical Pathway: the Perforant Path Input to the Dentate Area of the Hippocampal Formation. Exp. Brain Res 12 (1), 18–45. 5543199

[R165] London Z, and Albers JW (2007). Toxic Neuropathies Associated with Pharmaceutic and Industrial Agents. Neurol. Clin 25 (1), 257–276. doi:10.1016/j.ncl.2006.10.001 17324727

[R166] Lynch MA (2004). Long-term Potentiation and Memory. Physiol. Rev 84 (1), 87–136. doi:10.1152/physrev.00014.2003 14715912

[R167] Mack CM, Lin BJ, Turner JD, Johnstone AFM, Burgoon LD, and Shafer TJ (2014). Burst and Principal Components Analyses of MEA Data for 16 Chemicals Describe at Least Three Effects Classes. Neurotoxicology 40, 75–85. doi:10.1016/j.neuro.2013.11.008 24325902

[R168] Maffei L, and Fiorentini A (1986). Generator Sources of the Pattern ERG in Man and Animals. Front. Clin. Neurosci 3, 101–116.

[R169] Mallik A, and Weir A (2005). Nerve Conduction Studies: Essentials and Pitfalls in Practice. J. Neurol. Neurosurg. Psychiatry 76 (Suppl. 2), ii23–ii31. doi:10.1136/jnnp.2005.069138 15961865 PMC1765692

[R170] Manalis RS, and Cooper GP (1975). Evoked Transmitter Release Increased by Inorganic Mercury at Frog Neuromuscular junction. Nature 257 (5528), 690–691. doi:10.1038/257690a0 241937

[R171] Manalis RS, and Cooper GP (1973). Presynaptic and Postsynaptic Effects of lead at the Frog Neuromuscular junction. Nature 243 (5406), 354–356. doi:10.1038/243354a0 4542814

[R172] Marmont G (1949). Studies on the Axon Membrane. I. A New Method. J. Cel. Comp. Physiol 34 (3), 351–382. doi:10.1002/jcp.1030340303 15406358

[R173] Mattsson J, Albee RR, and Brandt LM (1984). H-reflex Waveform and Latency Variability in Rats. Fundam. Appl. Toxicol 4 (6), 944–948. doi:10.1016/0272-0590(84)90232-x 6519374

[R174] Mattsson J, and Albee R (1988). Sensory Evoked Potentials in Neurotoxicology. Neurotoxicology and Teratology 10 (5), 435–443. doi:10.1016/0892-0362(88)90005-0 3073306

[R175] Mattsson JL, Albee RR, and Eisenbrandt DL (1989a). Neurological Approach to Neurotoxicological Evaluation in Laboratory Animals. J. Am. Coll. Toxicol 8 (2), 271–286. doi:10.3109/10915818909019552

[R176] Mattsson JL, Albee RR, and Gorzinski SJ (1989b). Similarities of Toluene and *O*-Cresol Neuroexcitation in Rats. Neurotoxicology and Teratology 11 (1), 71–75. doi:10.1016/0892-0362(89)90088-3 2818714

[R177] Mattsson JL, Boyes WK, and Ross JF (1992). “Incorporating Evoked Potentials into Neurotoxicity Test Schemes,” in Neurotoxicology Editors Tilson Hand Mitchell C (New York: Raven Press), 125–145.

[R178] Mattsson JL, Eisenbrandt DL, and Albee RR (1990). Screening for Neurotoxicity: Complementarity of Functional and Morphologic Techniques. Toxicol. Pathol 18 (11 Pt 2), 115–127. doi:10.1177/019262339001800117 2195632

[R179] Maurer K, Bostock H, and Koltzenburg M (2007). A Rat *In Vitro* Model for the Measurement of Multiple Excitability Properties of Cutaneous Axons. Clin. Neurophysiol 118 (11), 2404–2412. doi:10.1016/j.clinph.2007.08.009 17897875

[R180] McNeil CJ, Butler JE, Taylor JL, and Gandevia SC (2013). Testing the Excitability of Human Motoneurons. Front. Hum. Neurosci 7, 152. doi:10.3389/fnhum.2013.00152 23630483 PMC3633937

[R181] Melnikov F, Geohagen BC, Gavin T, LoPachin RM, Anastas PT, Coish P, (2020). Application of the Hard and Soft, Acids and Bases (HSAB) Theory as a Method to Predict Cumulative Neurotoxicity. Neurotoxicology 79, 95–103. doi:10.1016/j.neuro.2020.04.009 32380191 PMC7369154

[R182] Meyer M, Dhamne SC, LaCoursiere CM, Tambunan D, Poduri A, and Rotenberg A (2016). Microarray Noninvasive Neuronal Seizure Recordings from Intact Larval Zebrafish. PLoS One 11 (6), e0156498. doi:10.1371/journal.pone.0156498 27281339 PMC4900632

[R183] Milan DJ, Jones IL, Ellinor PT, and MacRae CA (2006). *In Vivo* recording of Adult Zebrafish Electrocardiogram and Assessment of Drug-Induced QT Prolongation. Am. J. Physiology-Heart Circulatory Physiol 291 (1), H269–H273. doi:10.1152/ajpheart.00960.2005 16489111

[R184] Miller GW, Chandrasekaran V, Yaghoobi B, and Lein PJ (2018). Opportunities and Challenges for Using the Zebrafish to Study Neuronal Connectivity as an Endpoint of Developmental Neurotoxicity. Neurotoxicology 67, 102–111. doi:10.1016/j.neuro.2018.04.016 29704525 PMC6177215

[R185] Min B-K, Hämäläinen MS, and Pantazis D (2020). New Cognitive Neurotechnology Facilitates Studies of Cortical-Subcortical Interactions. Trends Biotechnol 38 (9), 952–962. doi:10.1016/j.tibtech.2020.03.003 32278504 PMC7442676

[R186] Mitzdorf U (1985). Current Source-Density Method and Application in Cat Cerebral Cortex: Investigation of Evoked Potentials and EEG Phenomena. Physiol. Rev 65 (1), 37–100. doi:10.1152/physrev.1985.65.1.37 3880898

[R187] Miura G, Wang MH, Ivers KM, and Frishman LJ (2009). Retinal Pathway Origins of the Pattern ERG of the Mouse. Exp. Eye Res 89 (1), 49–62. doi:10.1016/j.exer.2009.02.009 19250935 PMC2739005

[R188] Morgan DL, Little PB, Herr DW, Moser VC, Collins B, Herbert R, (2004). Neurotoxicity of Carbonyl Sulfide in F344 Rats Following Inhalation Exposure for up to 12 Weeks. Toxicol. Appl. Pharmacol 200 (2), 131–145. doi:10.1016/j.taap.2004.04.013 15476866

[R189] Mori A, Nodera H, Shibuta Y, Okita T, Bostock H, and Kaji R (2010). Threshold-dependent Effects on Peripheral Nerve *In Vivo* Excitability Properties in the Rat. Neurosci. Lett 468 (3), 248–253. doi:10.1016/j.neulet.2009.11.006 19900504

[R190] Muley N, Jain G, Singh JN, and Sharma SS (2009). Historical Events in Electrophysiology. Curr. Res. Inf. Pharmaceuticals Sci. (Crips) 10 (1), 2–8.

[R191] Müller J, Ballini M, Livi P, Chen Y, Radivojevic M, Shadmani A, (2015). High-resolution CMOS MEA Platform to Study Neurons at Subcellular, Cellular, and Network Levels. Lab. Chip 15 (13), 2767–2780. doi:10.1039/c5lc00133a 25973786 PMC5421573

[R192] Mwanza J-C, Finley D, Spivey CL, Graff JE, and Herr DW (2008). Depression of the Photic after Discharge of Flash Evoked Potentials by Physostigmine, Carbaryl and Propoxur, and the Relationship to Inhibition of Brain Cholinesterase. Neurotoxicology 29 (1), 87–100. doi:10.1016/j.neuro.2007.09.004 17950890

[R193] Nachtman JP, and Couri D (1984). An Electrophysiological Study of 2-hexanone and 2,5-hexanedione Neurotoxicity in Rats. Toxicol. Lett 23 (2), 141–145. doi:10.1016/0378-4274(84)90118-8 6506088

[R194] Nanda P, Morris A, Kelemen J, Yang J, and Wiest MC (2020). Evoked Frontal and Parietal Field Potential Signatures of Target Detection and Response Inhibition in Rats Performing an Equiprobable Auditory Go/no-Go Task. Eneuro 7 (1). doi:10.1523/ENEURO.0055-19.2019 PMC6944478 31767572

[R195] Narahashi T, and Haas HG (1967). DDT: Interaction with Nerve Membrane Conductance Changes. Science 157 (3795), 1438–1440. doi:10.1126/science.157.3795.1438 6037859

[R196] Narahashi T, and Haas HG (1968). Interaction of DDT with the Components of Lobster Nerve Membrane Conductance. J. Gen. Physiol 51 (2), 177–198. doi:10.1085/jgp.51.2.177 5641634 PMC2201121

[R197] Narahashi T, and Yamasaki T (1960). Mechanism of Increase in Negative After-Potential by Dicophanum (DDT) in the Giant Axons of the Cockroach*. J. Physiol 152 (1), 122–140. doi:10.1113/jphysiol.1960.sp006475 14426012 PMC1363301

[R198] Nasu S, Misawa S, Nakaseko C, Shibuya K, Isose S, Sekiguchi Y, (2014). Bortezomib-induced Neuropathy: Axonal Membrane Depolarization Precedes Development of Neuropathy. Clin. Neurophysiol 125 (2), 381–387. doi:10.1016/j.clinph.2013.07.014 23973385

[R199] National.Research.Council (2007). Toxicity Testing in the 21st Century: A Vision and a Strategy Washington, D.C.: National Academies Press, 0309109922.

[R200] Neher E, and Sakmann B (1976). Single-channel Currents Recorded from Membrane of Denervated Frog Muscle Fibres. Nature 260 (5554), 799–802. doi:10.1038/260799a0 1083489

[R201] Nicoll RA (2017). A Brief History of Long-Term Potentiation. Neuron 93 (2), 281–290. doi:10.1016/j.neuron.2016.12.015 28103477

[R202] Nieh EH, Kim S-Y, Namburi P, and Tye KM (2013). Optogenetic Dissection of Neural Circuits Underlying Emotional Valence and Motivated Behaviors. Brain Res 1511, 73–92. doi:10.1016/j.brainres.2012.11.001 23142759 PMC4099056

[R203] Nobili L (1828). Comparaison entre les deux galvanometres les plus sensibles, la grenouille et le moltiplicateur a deux aiguilles, suivie de quelques resultats noveaux. Ann. de Chim. de Physique 38, 225–245.

[R204] Nodera H, and Kaji R (2006). Nerve Excitability Testing and its Clinical Application to Neuromuscular Diseases. Clin. Neurophysiol 117 (9), 1902–1916. doi:10.1016/j.clinph.2006.01.018 16631406

[R205] Nodera H, and Rutkove SB (2012). Accommodation to Hyperpolarizing Currents: Differences between Motor and Sensory Nerves in Mice. Neurosci. Lett 518 (2), 111–116. doi:10.1016/j.neulet.2012.04.065 22579720

[R206] Nunez P (1995). Neocortical Dynamics and Human EEG Rhythms New York: Oxford University Press.

[R207] Nylén P, Hagman M, and Johnson A-C (1994). Function of the Auditory and Visual Systems, and of Peripheral Nerve, in Rats after Long-Term Combined Exposure to N-Hexane and Methylated Benzene Derivatives. I. Toluene. Pharmacol. Toxicol 74 (2), 116–123. doi:10.1111/j.1600-0773.1994.tb01085.x 8190699

[R208] Obergrussberger A, Bru ggemann A, Goetze TA, Rapedius M, Haarmann C, Rinke I, (2016). Automated Patch Clamp Meets High-Throughput Screening: 384 Cells Recorded in Parallel on a Planar Patch Clamp Module. J. Lab. Automation 21 (6), 779–793. doi:10.1177/2211068215623209 26702021

[R209] Obergrussberger A, Goetze TA, Brinkwirth N, Becker N, Friis S, Rapedius M, (2018). An Update on the Advancing High-Throughput Screening Techniques for Patch Clamp-Based Ion Channel Screens: Implications for Drug Discovery. Expert Opin. Drug Discov 13 (3), 269–277. doi:10.1080/17460441.2018.1428555 29343120

[R210] Organisation for Economic Co-Operation and Development (2016). Guidance Document for the Use of Adverse Outcome Pathways in Developing Integrated Approaches to Testing and Assessment (IATA), Series on Testing & Assessment Paris, France: OECD Publishing.

[R211] Overton E (1902). Beiträge zur allgemeinen Muskel- und Nervenphysiologie. Pflüger, Arch 92, 346–386. doi:10.1007/bf01659816

[R212] Panayiotopoulos CP, and Chroni E (1996). F-waves in Clinical Neurophysiology: a Review, Methodological Issues and Overall Value in Peripheral Neuropathies. Electroencephalography Clin. Neurophysiology/Electromyography Mot. Control 101 (5), 365–374. doi:10.1016/0924-980x(96)95635-0 8913188

[R213] Peng Y, Mittermaier FX, Planert H, Schneider UC, Alle H, and Geiger JRP (2019). High-throughput Microcircuit Analysis of Individual Human Brains through Next-Generation Multineuron Patch-Clamp. Elife 8, e48178. doi:10.7554/eLife.48178 31742558 PMC6894931

[R214] Piccolino M (1998). Animal Electricity and the Birth of Electrophysiology: the Legacy of Luigi Galvani. Brain Res. Bull 46 (5), 381–407. doi:10.1016/s0361-9230(98)00026-4 9739001

[R215] Polich J (2007). Updating P300: an Integrative Theory of P3a and P3b. Clin. Neurophysiol 118 (10), 2128–2148. doi:10.1016/j.clinph.2007.04.019 17573239 PMC2715154

[R216] Pollard KJ, Bolon B, and Moore MJ (2021). Comparative Analysis of Chemotherapy-Induced Peripheral Neuropathy in Bioengineered Sensory Nerve Tissue Distinguishes Mechanistic Differences in Early-Stage Vincristine-, Cisplatin-, and Paclitaxel-Induced Nerve Damage. Toxicol. Sci 180 (1), 76–88. doi:10.1093/toxsci/kfaa186 33410881 PMC7916732

[R217] Poon E, Powers BE, McAlonan RM, Ferguson DC, and Schantz SL (2011). Effects of Developmental Exposure to Polychlorinated Biphenyls And/or Polybrominated Diphenyl Ethers on Cochlear Function. Toxicol. Sci 124 (1), 161–168. doi:10.1093/toxsci/kfr214 21873374 PMC3196655

[R218] Racine RJ, Gartner JG, and McIntyre Burnham W (1972). Epileptiform Activity and Neural Plasticity in Limbic Structures. Brain Res 47 (1), 262–268. doi:10.1016/0006-8993(72)90268-5 4641271

[R219] Racine RJ, Milgram NW, and Hafner S (1983). Long-term Potentiation Phenomena in the Rat Limbic Forebrain. Brain Res 260 (2), 217–231. doi:10.1016/0006-8993(83)90676-5 6299454

[R220] Radnikow G, Günter RH, Marx M, and Feldmeyer D (2011). “Morpho-functional Mapping of Cortical Networks in Brain Slice Preparations Using Paired Electrophysiological Recordings,” in Neuronal Network Analysis. Neuromethods Editors Fellin T and Halassa M (New YorK, N.Y.: Humana Press), 405–431. 978-1-61779-632-6978-1-61779-633-3. doi:10.1007/7657_2011_14

[R221] Rebert CS (1983). Multisensory Evoked Potentials in Experimental and Applied Neurotoxicology. Neurobehav Toxicol. Teratol 5 (6), 659–671. 6686861

[R222] Rebert CS, and Sorenson SS (1983). Concentration-related Effects of Hexane on Evoked Responses from Brain and Peripheral Nerve of the Rat. Neurobehav Toxicol. Teratol 5 (1), 69–76. 6856011

[R223] Rebert CS, Pryor GT, and Frick MS (1984). Effects of Vincristine, Maytansine, Andcis-Platinum on Behavioral and Electrophysiological Indices of Neurotoxicity in the Rat. J. Appl. Toxicol 4 (6), 330–338. doi:10.1002/jat.2550040610 6542922

[R224] Renault R, Sukenik N, Descroix S, Malaquin L, Viovy J-L, Peyrin J-M, (2015). Combining Microfluidics, Optogenetics and Calcium Imaging to Study Neuronal Communication *In Vitro*. PLoS One 10 (4), e0120680. doi:10.1371/journal.pone.0120680 25901914 PMC4406441

[R225] Richmond JE, and Jorgensen EM (1999). One GABA and Two Acetylcholine Receptors Function at the *C. elegans* Neuromuscular junction. Nat. Neurosci 2 (9), 791–797. doi:10.1038/12160 10461217 PMC2585773

[R226] Robinette BL, Harrill JA, Mundy WR, and Shafer TJ (2011). *In Vitro* assessment of Developmental Neurotoxicity: Use of Microelectrode Arrays to Measure Functional Changes in Neuronal Network Ontogeny. Front. Neuroeng 4, 1. doi:10.3389/fneng.2011.00001 21270946 PMC3026483

[R227] Robinson AK, Venkatesh P, Boring MJ, Tarr MJ, Grover P, and Behrmann M (2017). Very High Density EEG Elucidates Spatiotemporal Aspects of Early Visual Processing. Sci. Rep 7 (1), 1–11. doi:10.1038/s41598-017-16377-3 29176609 PMC5701165

[R228] Ross JF (1989). Applications of Electrophysiology in a Neurotoxicity Battery. Toxicol. Ind. Health 5 (2), 221–230. doi:10.1177/074823378900500207 2728016

[R229] Ross JF, and Lawhorn GT (1990). ZPT-related Distal Axonopathy: Behavioral and Electrophysiologic Correlates in Rats. Neurotoxicology and Teratology 12 (2), 153–159. doi:10.1016/0892-0362(90)90128-y 2333068

[R230] Ruijten MWMM, Sallé HJA, and Kingma R (1993). Comparison of Two Techniques to Measure the Motor Nerve Conduction Velocity Distribution. Electroencephalography Clin. Neurophysiology/Evoked Potentials Section 89 (6), 375–381. doi:10.1016/0168-5597(93)90110-b 7507423

[R231] Sack AT, and Linden DEJ (2003). Combining Transcranial Magnetic Stimulation and Functional Imaging in Cognitive Brain Research: Possibilities and Limitations. Brain Res. Rev 43 (1), 41–56. doi:10.1016/s0165-0173(03)00191-7 14499461

[R232] Sandström J, Eggermann E, Charvet I, Roux A, Toni N, Greggio C, (2017). Development and Characterization of a Human Embryonic Stem Cell-Derived 3D Neural Tissue Model for Neurotoxicity Testing. Toxicol. Vitro 38, 124–135. doi:10.1016/j.tiv.2016.10.001 27729293

[R233] Sawai S, Kanai K, Nakata M, Hiraga A, Misawa S, Isose S, (2008). Changes in Excitability Properties Associated with Axonal Regeneration in Human Neuropathy and Mouse Wallerian Degeneration. Clin. Neurophysiol 119 (5), 1097–1105. doi:10.1016/j.clinph.2008.01.022 18342570

[R234] Sã¤nger J, Bechtold L, Schoofs D, Blaszkewicz M, and Wascher E (2014). The Influence of Acute Stress on Attention Mechanisms and its Electrophysiological Correlates. Front. Behav. Neurosci 8, 353. doi:10.3389/fnbeh.2014.00353 25346669 PMC4191471

[R235] Senzai Y, Fernandez-Ruiz A, and Buzsáki G (2019). Layer-specific Physiological Features and Interlaminar Interactions in the Primary Visual Cortex of the Mouse. Neuron 101 (3), 500–513. doi:10.1016/j.neuron.2018.12.009 30635232 PMC6367010

[R236] Serra J, Bostock H, and Navarro X (2010). Microneurography in Rats: a Minimally Invasive Method to Record Single C-Fiber Action Potentials from Peripheral Nerves *In Vivo*. Neurosci. Lett 470 (3), 168–174. doi:10.1016/j.neulet.2009.09.061 19800936

[R237] Shafer TJ (2019). Application of Microelectrode Array Approaches to Neurotoxicity Testing and Screening. Vitro Neuronal Networks 22, 275–297. doi:10.1007/978-3-030-11135-9_12 31073941

[R238] Shanes AM (1949). Electrical Phenomena in Nerve. J. Gen. Physiol 33 (1), 75–102. doi:10.1085/jgp.33.1.75 18139009 PMC2147141

[R239] Sharma AD, McCoy L, Jacobs E, Willey H, Behn JQ, Nguyen H, (2019). Engineering a 3D Functional Human Peripheral Nerve *In Vitro* Using the Nerve-On-A-Chip Platform. Sci. Rep 9 (1), 1–12. doi:10.1038/s41598-019-45407-5 31222141 PMC6586937

[R240] Shityakov S, and Förster CY (2018). Computational Simulation and Modeling of the Blood-Brain Barrier Pathology. Histochem. Cel Biol 149 (5), 451–459. doi:10.1007/s00418-018-1665-x 29721642

[R241] Sills RC, Morgan DL, Herr DW, Little PB, George NM, Thai Vu Ton TV, (2004). Contribution of Magnetic Resonance Microscopy in the 12-week Neurotoxicity Evaluation of Carbonyl Sulfide in Fischer 344 Rats. Toxicol. Pathol 32 (5), 501–510. doi:10.1080/01926230490493918 15603534

[R242] Simão D, Silva MM, Terrasso AP, Arez F, Sousa MFQ, Mehrjardi NZ, (2018). Recapitulation of Human Neural Microenvironment Signatures in iPSC-Derived NPC 3D Differentiation. Stem Cel Rep 11 (2), 552–564. doi:10.1016/j.stemcr.2018.06.020 PMC6094163 30057262

[R243] Singh AK, Touhara K, and Okamoto M (2019). Electrophysiological Correlates of Top-Down Attentional Modulation in Olfaction. Sci. Rep 9 (1), 4953. doi:10.1038/s41598-019-41319-6 30894641 PMC6426950

[R244] Singh G, Avasthi G, Khurana D, Whig J, and Mahajan R (1998). Neurophysiological Monitoring of Pharmacological Manipulation in Acute Organophosphate (OP) Poisoning. The Effects of Pralidoxime, Magnesium Sulphate and Pancuronium. Electroencephalography Clin. Neurophysiol 107 (2), 140–148. doi:10.1016/s0013-4694(98)00053-4 9751285

[R245] Sirenko O, Parham F, Dea S, Sodhi N, Biesmans S, Mora-Castilla S, (2019). Functional and Mechanistic Neurotoxicity Profiling Using Human iPSC-Derived Neural 3D Cultures. Toxicol. Sci 167 (1), 58–76. doi:10.1093/toxsci/kfy218 30169818 PMC6317428

[R246] Staicu CE, Jipa F, Axente E, Radu M, Radu BM, and Sima F (2021). Lab-on-a-Chip Platforms as Tools for Drug Screening in Neuropathologies Associated with Blood-Brain Barrier Alterations. Biomolecules 11 (6), 916. doi:10.3390/biom11060916 34205550 PMC8235582

[R247] Stålberg E, and Erdem H (2000). Nerve Conduction Studies. J. Neurol. Sci 17 (2), 1302–1664.

[R248] Stålberg E, and Sanders DB (1981). “Electrophysiological Tests of Neuromuscular Transmission,” in Clinical Neurophysiology Editors Stålberg E and Young R (London: Butterworths), 88–116.

[R249] Stålberg E, and Sonoo M (1994). Assessment of Variability in the Shape of the Motor Unit Action Potential, the “jiggle,” at Consecutive Discharges. Muscle & Nerve 17, 1135–1144. 7935520 10.1002/mus.880171003

[R250] Steinmetz NA, Aydin C, Lebedeva A, Okun M, Pachitariu M, Bauza M, (2020). Neuropixels 2.0: A Miniaturized High-Density Probe for Stable, Long-Term Brain Recordings. bioRxiv 1027, 358291. 10.1126/science.abf4588 PMC8244810 33859006

[R251] Steinmetz NA, Zatka-Haas P, Carandini M, and Harris KD (2019). Distributed Coding of Choice, Action and Engagement across the Mouse Brain. Nature 576 (7786), 266–273. doi:10.1038/s41586-019-1787-x 31776518 PMC6913580

[R252] Stolzberg D, Chrostowski M, Salvi RJ, and Allman BL (2012). Intracortical Circuits Amplify Sound-Evoked Activity in Primary Auditory Cortex Following Systemic Injection of Salicylate in the Rat. J. Neurophysiol 108 (1), 200–214. doi:10.1152/jn.00946.2011 22496535 PMC3434608

[R253] Stone NL, England TJ, and O’Sullivan SE (2019). A Novel Transwell Blood Brain Barrier Model Using Primary Human Cells. Front. Cel. Neurosci 13, 230. doi:10.3389/fncel.2019.00230 PMC6563620 31244605

[R254] Sung J-Y, Tani J, Park SB, Kiernan MC, and Lin CS-Y (2014). Early Identification of ‘acute-Onset’ Chronic Inflammatory Demyelinating Polyneuropathy. Brain 137 (8), 2155–2163. doi:10.1093/brain/awu158 24983276 PMC4610188

[R255] Sutton S, Braren M, Zubin J, and John ER (1965). Evoked-potential Correlates of Stimulus Uncertainty. Science 150 (3700), 1187–1188. doi:10.1126/science.150.3700.1187 5852977

[R256] Szymanski FD, Garcia-Lazaro JA, and Schnupp JWH (2009). Current Source Density Profiles of Stimulus-specific Adaptation in Rat Auditory Cortex. J. Neurophysiol 102 (3), 1483–1490. doi:10.1152/jn.00240.2009 19571199

[R257] Thompson SW, Davis LE, Kornfeld M, Hilgers RD, and Standefer JC (1984). Cisplatin Neuropathy. Clinical, Electrophysiologic, Morphologic, and Toxicologic Studies. Cancer 54 (7), 1269–1275. doi:10.1002/1097-0142(19841001)54:7<1269:aid-cncr2820540707>3.0.co;2-9 6088023

[R258] Timme N, Ma B, Linsenbardt DN, Cornwell E, Galbari T, and Lapish C, 2021. Compulsive Drinking Is Associated with Neural Activity Patterns Reflecting Diminished Behavioral Control and Enhanced Seeking Representations in Dorsal Medial Prefrontal Cortex. bioRxiv10.1038/s41467-022-31731-4 PMC9271071 35810193

[R259] Trachtenberg JT, Chen BE, Knott GW, Feng G, Sanes JR, Welker E, (2002). Long-term *In Vivo* Imaging of Experience-dependent Synaptic Plasticity in Adult Cortex. Nature 420 (6917), 788–794. doi:10.1038/nature01273 12490942

[R260] Tucker KJ, Tuncer M, and Türker KS (2005). A Review of the H-Reflex and M-Wave in the Human Triceps Surae. Hum. Movement Sci 24 (5–6), 667–688. doi:10.1016/j.humov.2005.09.010 16337293

[R261] United States Environmental Protection Agency (1998a). “Health Effects Test Guidelines. OPPTS 870.6850. Peripheral Nerve Function,” in Office of Prevention Editor P. A. T.S (Washington, D.C., 1–9.

[R262] United States Environmental Protection Agency (1998b). “Health Effects Test Guidelines. OPPTS 870.6855. Neurophysiology: Sensory Evoked Potentials,” in Office of Prevention Editor P. A. T.S (Washington, D.C., 1–14.

[R263] Urai AE, Doiron B, Leifer AM, and Churchland AK, 2021. Large-scale Neural Recordings Call for New Insights to Link Brain and Behavior. arXiv: 2103.14662. 10.1038/s41593-021-00980-9 34980926

[R264] van Der Helm MW, Van Der Meer AD, Eijkel JCT, van den Berg A, and Segerink LI (2016). Microfluidic Organ-On-Chip Technology for Blood-Brain Barrier Research. Tissue Barriers 4 (1), e1142493. doi:10.1080/21688370.2016.1142493 27141422 PMC4836466

[R265] Verkhratsky A, Krishtal OA, and Petersen OH (2006). From Galvani to Patch Clamp: the Development of Electrophysiology. Pflugers Arch. - Eur. J. Physiol 453 (3), 233–247. doi:10.1007/s00424-006-0169-z 17072639

[R266] Verkhratsky A, and Parpura V (2014). History of Electrophysiology and the Patch Clamp, Patch-Clamp Methods and Protocols Springer, 1–19. doi:10.1007/978-1-4939-1096-0_1 25023299

[R267] Verleger R (1988). Event-related Potentials and Cognition: A Critique of the Context Updating Hypothesis and an Alternative Interpretation of P3. Behav. Brain Sci 11 (3), 343–356. doi:10.1017/s0140525x00058015

[R268] Victor Nadler J, and Cuthbertson GJ (1980). Kainic Acid Neurotoxicity toward Hippocampal Formation: Dependence on Specific Excitatory Pathways. Brain Res 195 (1), 47–56. doi:10.1016/0006-8993(80)90865-3 6249441

[R269] Vogel EK, and Luck SJ (2000). The Visual N1 Component as an index of a Discrimination Process. Psychophysiology 37 (2), 190–203. doi:10.1111/1469-8986.3720190 10731769

[R270] Ward NL, and Lamanna JC (2004). The Neurovascular Unit and its Growth Factors: Coordinated Response in the Vascular and Nervous Systems. Neurol. Res 26 (8), 870–883. doi:10.1179/016164104x3798 15727271

[R271] Wijers AA, Otten LJ, Feenstra S, Mulder G, and Mulder LJM (1989). Brain Potentials during Selective Attention, Memory Search, and Mental Rotation. Psychophysiology 26 (4), 452–467. doi:10.1111/j.1469-8986.1989.tb01951.x 2798695

[R272] Wood CC (1982). Application of Dipole Localization Methods to Source Identification of Human Evoked Potentials. Ann. NY Acad. Sci 388 (1), 139–155. doi:10.1111/j.1749-6632.1982.tb50789.x 6953865

[R273] Xie C, Liu J, Fu T-M, Dai X, Zhou W, and Lieber CM (2015). Three-dimensional Macroporous Nanoelectronic Networks as Minimally Invasive Brain Probes. Nat. Mater 14 (12), 1286–1292. doi:10.1038/Nmat4427 26436341

[R274] Yang D, He F, and Li T (2001). Repetitive Nerve Stimulation and Stimulation Single Fiber Electromyography Studies in Rats Intoxicated with Single or Mixed Insecticides. Toxicology 161 (1–2), 111–116. doi:10.1016/s0300-483x(01)00339-0 11295260

[R275] Ylä-Outinen L, Heikkilä J, Skottman H, Suuronen R, Äänismaa R, and Narkilahti S (2010). Human Cell-Based Micro Electrode Array Platform for Studying Neurotoxicity. Front. Neuroengineering 3, 111. 10.3389/fneng.2010.00111 PMC2955435 20953240

[R276] Zhuang P, Sun AX, An J, Chua CK, and Chew SY (2018). 3D Neural Tissue Models: From Spheroids to Bioprinting. Biomaterials 154, 113–133. doi:10.1016/j.biomaterials.2017.10.002 29120815

[R277] Zotova EG, and Arezzo JC (2013). Non-Invasive Evaluation of Nerve Conduction in Small Diameter Fibers in the Rat. Physiol. J 2013, 254789. doi:10.1155/2013/254789 23580940 PMC3620683

[R278] Zotova EG, Schaumburg HH, Raine CS, Cannella B, Tar M, Melman A, (2008). Effects of Hyperglycemia on Rat Cavernous Nerve Axons: a Functional and Ultrastructural Study. Exp. Neurol 213 (2), 439–447. doi:10.1016/j.expneurol.2008.07.009 18687329 PMC2586390

[R279] Zwartsen A, Hondebrink L, and Westerink RH (2018). Neurotoxicity Screening of New Psychoactive Substances (NPS): Effects on Neuronal Activity in Rat Cortical Cultures Using Microelectrode Arrays (MEA). Neurotoxicology 66, 87–97. doi:10.1016/j.neuro.2018.03.007 29572046

